# Actin dynamics provides membrane tension to merge fusing vesicles into the plasma membrane

**DOI:** 10.1038/ncomms12604

**Published:** 2016-08-31

**Authors:** Peter J. Wen, Staffan Grenklo, Gianvito Arpino, Xinyu Tan, Hsien-Shun Liao, Johanna Heureaux, Shi-Yong Peng, Hsueh-Cheng Chiang, Edaeni Hamid, Wei-Dong Zhao, Wonchul Shin, Tuomas Näreoja, Emma Evergren, Yinghui Jin, Roger Karlsson, Steven N. Ebert, Albert Jin, Allen P. Liu, Oleg Shupliakov, Ling-Gang Wu

**Affiliations:** 1 National Institute of Neurological Disorders and Stroke, 35 Convent Drive, Building 35, Room 2B-1012, Bethesda, Maryland 20892, USA; 2 Center of Excellence in Developmental Biology, Department of Neuroscience, Karolinska Institutet, S-171 77 Stockholm, Sweden; 3 Department of Cell Biology, WGI, Stockholm University, 106 91 Stockholm, Sweden; 4 Department of Mechanical Engineering, University of Michigan, Ann Arbor, Michigan 48109, USA; 5 National Institute of Biomedical Imaging and Bioengineering (NIBIB), Bethesda, Maryland 20892, USA; 6 Burnett School of Biomedical Sciences, College of Medicine, University of Central Florida, 6900 Lake Nona Boulevard, Orlando, Florida 32827, USA; 7 Institute of Translational Biomedicine, St. Petersburg State University, St. Petersburg 199034, Russia

## Abstract

Vesicle fusion is executed via formation of an Ω-shaped structure (Ω-profile), followed by closure (kiss-and-run) or merging of the Ω-profile into the plasma membrane (full fusion). Although Ω-profile closure limits release but recycles vesicles economically, Ω-profile merging facilitates release but couples to classical endocytosis for recycling. Despite its crucial role in determining exocytosis/endocytosis modes, how Ω-profile merging is mediated is poorly understood in endocrine cells and neurons containing small ∼30–300 nm vesicles. Here, using confocal and super-resolution STED imaging, force measurements, pharmacology and gene knockout, we show that dynamic assembly of filamentous actin, involving ATP hydrolysis, N-WASP and formin, mediates Ω-profile merging by providing sufficient plasma membrane tension to shrink the Ω-profile in neuroendocrine chromaffin cells containing ∼300 nm vesicles. Actin-directed compounds also induce Ω-profile accumulation at lamprey synaptic active zones, suggesting that actin may mediate Ω-profile merging at synapses. These results uncover molecular and biophysical mechanisms underlying Ω-profile merging.

## Abstract (web summary)

As vesicles fuse to the plasma membrane, they form intermediate Ω-shaped structures followed by either closure of the pore or full merging with the plasma membrane. Here Wen *et al*. show that dynamic actin assembly provides membrane tension to promote Ω merging in neuroendocrine cells and synapses.

## Graphical abstract

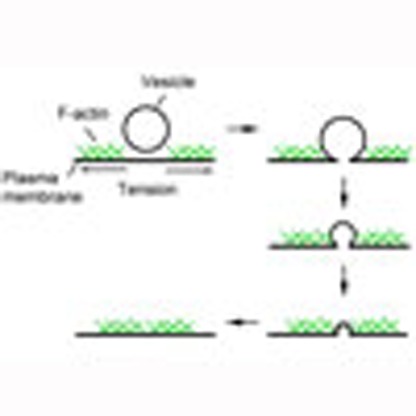

Vesicle fusion releases vesicular contents such as hormones, peptides and transmitters, to mediate many biological processes crucial to an animal's life, such as stress responses, mood changes, synaptic transmission, neuronal network activity, and immune responses^1^,^2^,^3^,^4^. It is executed via formation of an Ω-shape intermediate structure, termed Ω-profile, at the plasma membrane for releasing contents, followed by closure (called kiss-and-run) or merging of the Ω-profile into the plasma membrane (called full fusion)^1^,^2^,^3^,^4^. Ω-profile closure limits vesicular content release and cargo delivery, but recycles vesicles economically^1^,^2^. In contrast, Ω-profile merging allows for rapid, complete content release and cargo delivery, but couples exocytosis to classical endocytosis involving membrane invagination, Ω-profile formation and fission, for retrieving merged vesicles^1^,^2^,^3^. In other words, Ω-profile merging defines the mode of fusion (full fusion versus kiss-and-run) and the mode of endocytosis (classical endocytosis versus kiss-and-run). Despite these fundamental roles, the mechanism underlying Ω-profile merging is unclear in endocrine cells and neurons where vesicles are ≤∼300 nm and fusion takes place rapidly after calcium influx. Ω-profile merging is often assumed to be a passive, automatic process with no energy consumption once fusion pore opens in neurons and neuroendocrine cells.

Ω-profile merging has recently been studied in secretory cells containing extremely large vesicles (∼1-5 μm), such as in *Xenopus* oocytes^5^, human endothelial cells, lacrimal epithelial acinar cells^6^, parotid and pancreatic acinar cells^7^,^8^, and type II pneumocytes^9^, where Ω-profile merging and release take extremely long time (tens of seconds to tens of minutes) and release is not as tightly coupled to calcium influx as in neuroendocrine cells and neurons (reviewed in ref. 10). These studies suggest that cytoskeletal filamentous actin (F-actin) may coat the fusing Ω-profile in a few to tens of seconds after fusion, which may mediate two seemingly opposite functions: to compress the fusing Ω-profile and thus to merge the Ω-profile with the plasma membrane, or to hold the Ω-profile from collapsing into the plasma membrane. Whether and which of these mechanisms apply to endocrine cells and neurons containing smaller vesicles remain unclear, mostly due to difficulty of detecting the very transient process of Ω-profile merging in smaller vesicles.

In the present work, we overcame the difficulty of detecting Ω-profile merging in smaller vesicles by confocal imaging and super-resolution stimulated emission depletion (STED) imaging in neuroendocrine chromaffin cells^11^ and by electron microscopy (EM) at lamprey synapses. Combined with pharmacological tools and gene knockout (KO), we found that dynamic assembly of cytoskeletal F-actin is necessary for Ω-profile merging in chromaffin cells. With imaging and manipulations of plasma membrane mechanical forces, our results suggest that F-actin mediates Ω-profile merging by providing sufficient tension at the plasma membrane to shrink the Ω-profile. We also found that the F-actin assembly pathway including hydrolysis of the energy molecule ATP, neuronal Wiskott–Aldrich syndrome protein (N-WASP) and formin that activate F-actin assembly participates in mediating Ω-profile merging. F-actin-mediated Ω-profile merging is probably applicable to synapses, because block of F-actin led to accumulation of Ω-profiles at the active zone of lamprey giant synapses. These results uncover novel molecular and biophysical mechanisms underlying Ω-profile merging in neuroendocrine cells and neurons, which mediates full fusion and couples exocytosis to classical endocytosis.

## Results

### Imaging in conditions that facilitate Ω-profile merging

**Figure 1:**
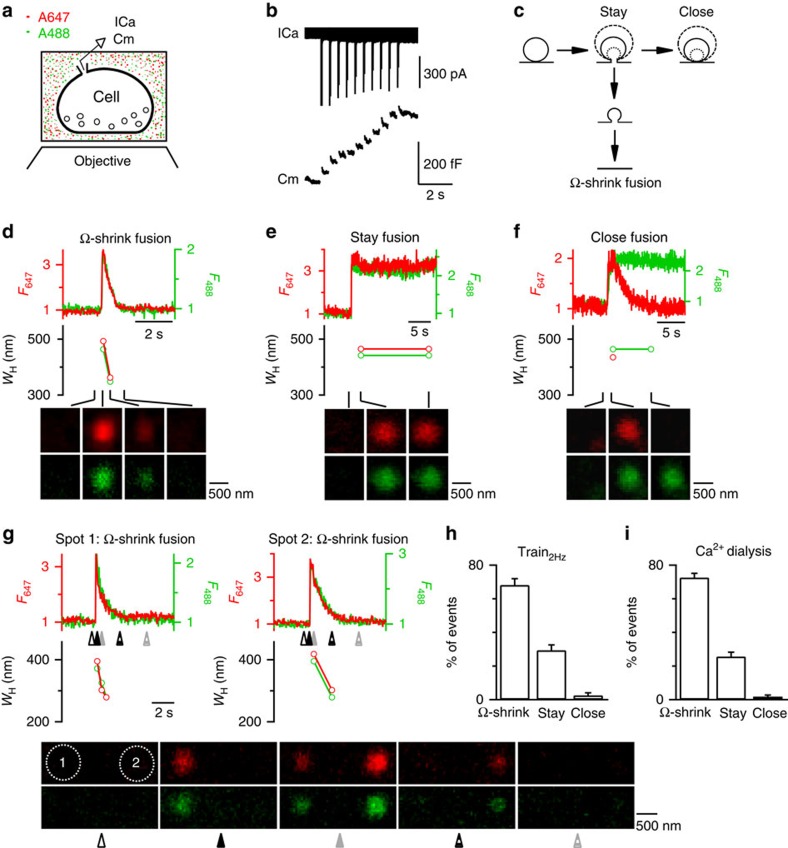
Train_2Hz_ induces three fusion forms with Ω-shrink as the dominant form in control chromaffin cells. (**a**) Schematic drawing of a cell on the coverslip bathed with a solution containing A647 (red) and A488 (green). ICa and membrane capacitance (Cm) are whole-cell recorded and the cell bottom is imaged confocally. (**b**) Sampled ICa and the Cm change induced by Train_2Hz_. (**c**) A schematic diagram showing the Ω-exo-endocytosis model, where the fusion-generated Ω-profile may stay at the site (stay fusion), close the pore (close fusion) or shrink until undetectable (Ω-shrink fusion). For stay and close fusion, the Ω-profile may change in sizes (drawing of different sizes of Ω-profiles). Examples of Ω-shrink (**d**), stay (**e**) and close (**f**) fusion induced by Train_2Hz_: *F*_647_ (red), *F*_488_ (green), *W*_H_ measured from A647 (red) or A488 (green) spot and sampled images (4–15 frame average, A647, red; A488, green, lower) at times indicated (lines) are plotted versus time. Images were collected every 20–40 ms at the confocal cell-bottom setting (A647 excited strongly; A488 excited weakly). *F*_647_ or *F*_488_ was normalized to its mean background value before spots appeared. *W*_H_ was taken before the spot was too dim to measure. The images were obtained at the cell bottom. These settings apply to all similar plots in Figs 1, 2, 3, 4. (**g**) An example showing that Ω-shrink fusion is the majority during Train_2Hz_ in control: two neighbouring spots (1 and 2) underwent Ω-shrink fusion. Sampled images (lower) were taken at times indicated by triangles below traces (upper). (**h**,**i**) Percentages (mean+s.e.m.) of Ω-shrink, stay and close fusion spots induced by Train_2Hz_ (*n*=18 cells; total spot number: 192; **h**) or whole-cell calcium dialysis (1.5 μM, *n*=23 cells, total spot number: 399; **i**) in control conditions.

We used a recently developed technique to image Ω-profile merging in live, primary-cultured bovine adrenal chromaffin cells containing ∼300 nm dense-core vesicles in a bath solution containing membrane-impermeable Alexa Fluor 647 (A647) and Alexa Fluor 488 (A488) (Fig. 1a)^11^. Cells were voltage clamped at −80 mV and stimulated with 10 pulses of 50 ms depolarization to +10 mV at 2 Hz (Train_2Hz_). The resulting calcium current (ICa) and capacitance increases that reflect fusion were whole-cell recorded (Fig. 1b). During and within 3 s after Train_2Hz_, A647 and A488 spots reflecting dye-filled fusion-generated Ω-profiles appeared^11^ (Fig. 1c–f).

Consistent with our previous findings using a 1 s depolarization^11^, Ω-profiles may change in seven patterns (Supplementary Fig. 1a), which are grouped into three categories (Fig. 1c) as follows: (1) the Ω-profile shrinks until undetectable, leading to Ω-profile merging with the plasma membrane, termed Ω-shrink fusion; (2) the Ω-profile is maintained while its size may remain unchanged, enlarged or shrink to some extent, termed stay fusion; and (3) the stay fusion is followed by pore closure, termed close fusion^11^. With confocal imaging of A647 and A488 (every 20–40 ms) at the cell bottom using strong and weak excitation, respectively (confocal A647_strong_/A488_weak_ setting)^11^, Ω-shrink was identified as parallel decay of A647 and A488 spot fluorescence (*F*_647_ and *F*_488_, respectively) to baseline, whereas A647 (or A488) spot size, measured from the full-width half-maximum (*W*_H_), reduced until below confocal resolution (Fig. 1d and Supplementary Fig. 1b,c; for detail, see ref. 11). Stay fusion was identified as the persistence of the A647 and A488 spot for >30 s (our recording time; Fig. 1e). During stay fusion, the spot's *W*_H_ may remain unchanged, reduce to some extent or enlarge, whereas *F*_647_ and *F*_488_ changed in parallel (Supplementary Fig. 1d; for detail, see ref. 11). Close fusion was reflected as stay fusion followed by bleaching of the spot *F*_647_ to baseline, but no change of *F*_488_ signal (Fig. 1f and Supplementary Fig. 1e), because pore closure prevented exchange of bleached A647 (due to strong excitation) with fluorescent A647 in the bath, resulting in the decay of the spot *F*_647_, but not the weakly excited *F*_488_ (ref. 11). These three categories were confirmed with several other methods, including high-resolution STED microscopy (for example, Supplementary Fig. 1c)^11^.

With 1 s depolarization, we previously found that Ω-shrink fusion percentage increases as ICa decreases^11^. To search for stimulation protocols that facilitate Ω-shrink fusion, we decreased ICa charge by reducing the depolarization duration to 50 ms. To induce more granule fusion, we used 10 pulses of 50 ms depolarization at 2 Hz (Train_2Hz_). With Train_2Hz_, 68±4% of fusion events were Ω-shrink fusion, 29±3% were stay fusion and 3±2% were close fusion (Fig. 1g,h; *n*=18 cells, total fusion spots: 192; ±indicates s.e. throughout this study). Similarly, whole-cell dialysis of 1.5 μM free calcium induced mostly Ω-shrink fusion (Fig. 1i). Throughout this study, we used Train_2Hz_ to induce fusion if not mentioned otherwise.

### Dynamic F-actin assembly mediates Ω-profile merging

**Figure 2:**
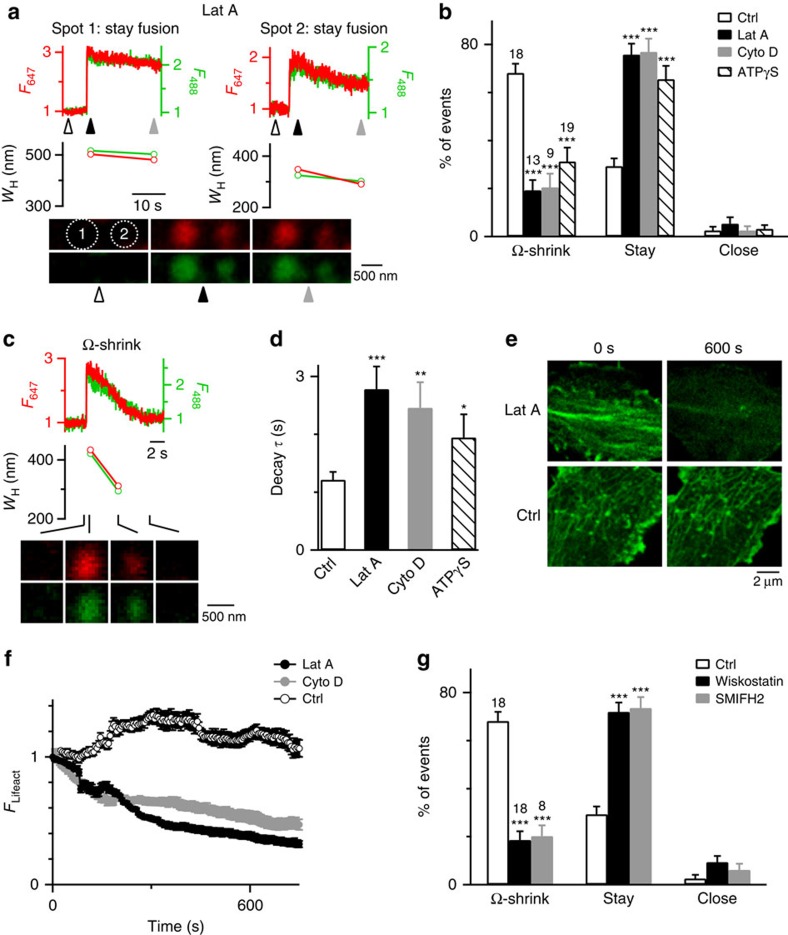
Block of F-actin inhibits Ω-shrink fusion but promotes stay fusion. (**a**) An example showing more spots (two neighbouring spots, 1 and 2) undergoing stay fusion during Train_2Hz_ in the presence of Lat A (3 μM, bath). Sampled confocal images were taken at times indicated by triangles. (**b**) Percentages (mean+s.e.m.) of Train_2Hz_-induced spots undergoing Ω-shrink, stay or close fusion in control (Ctrl, same as Fig. 1h, *n*=18 cells) or in the presence of 3 μM Lat A (*n*=13 cells; total spot number: 150; ***P<0.001), 4 μM Cyto D (*n*=9 cells; 91 spots; ***P<0.001) or ATPγS (2 mM, replacing 2 mM ATP in the pipette; *n*=19 cells, 213 spots; ***P<0.001). Cell numbers are also shown on top of the bar (apples to other similar bar graphs). Error bars indicate s.e.m. (**c**) A A647/A488-filled spot undergoing Ω-shrink fusion in the presence of Lat A: *F*_647_ (red), *F*_488_ (green), *W*_H_ measured from A647 (red) or A488 (green) spot and sampled images at times indicated (lines) are plotted versus time. Images were obtained at the cell bottom with confocal microscopy. Shrinking rate is slower compared with control in Fig. 1d,g. (**d**) *F*_647_ decay *τ* (mean+s.e.m.) for Ω-shrink fusion in control (*n*=130 spots), in the presence of Lat A (*n*=33 spots, ***P<0.001), Cyto D (*n*=20 spots, **P=0.0016) or ATP-γS (*n*=70 spots, *P=0.0363). (**e**) Sampled Lifeact images at the cell bottom before (0 s, left) and 600 s after (right) application of Lat A (3 μM) or a control (Ctrl) solution. (**f**) Lifeact-TagGFP2 fluorescence (*F*_Lifeact_, mean±s.e.m.) at the cell bottom before (time 0) and during application of a control solution (Ctrl, 11 cells), Lat A (3 μM, 6 cells) or Cyto D (4 μM, 6 cells). *F*_Lifeact_ was normalized to the value at time 0. (**g**) Percentages (mean+s.e.m.) of Train_2Hz_-induced spots undergoing Ω-shrink, stay or close fusion in control (Ctrl, same as Fig. 1h) or in the presence of 10 μM wiskostatin (*n*=18 cells; total spot number: 175; ***P<0.001) or 25 μM SMIFH2 (*n*=8 cells; 74 spots; ***P<0.001) in the bath solution. Statistical test for **b**, **d** and **g** was Student's *t*-test.

Application of latrunculin A (Lat A, 3 μM, bath), which disrupted F-actin polymerization^12^, reduced Ω-shrink fusion from 68 to 19% (13 cells), increased stay fusion from 29 to 76%, but did not affect close fusion (Fig. 2a,b). The remaining Ω-shrink fusion in the presence of Lat A had a longer *F*_647_ decay time constant (*τ*, 2.8 s, for example, Fig. 2c; summarized in Fig. 2d) than in control (1.2 s, for example, Fig. 1d,g), indicating a reduction in Ω-profile shrinking rate. Thus, Lat A slows down or blocks Ω-profile shrinking. The percentage decrease in Ω-shrink fusion was mirrored by the increased stay fusion percentage (Fig. 2b), indicating that Lat A converts Ω-shrink to stay fusion. Similarly, another actin polymerization inhibitor cytochalasin D (Cyto D, 4 μM, bath)^13^,^14^ inhibited Ω-shrink and increased stay fusion, suggesting that actin polymerization mediates Ω-profile merging (Fig. 2b,d and Supplementary Fig. 2). As expected^12^,^13^,^14^, Lat A and Cyto D significantly reduced F-actin labelled with overexpressed Lifeact-TagGFP2 (Lifeact) at the cell bottom where we examined fusion (Fig. 2e,f).

Similar to Lat A/Cyto D, wiskostatin (10 μM), which inhibits N-WASP-mediated, Arp2/3-dependent F-actin assembly^15^, and SMIFH2 (25 μM), which blocks formin-profilin binding involved in formin-dependent F-actin assembly^16^, inhibited Ω-shrink and promoted stay fusion (Fig. 2g). In contrast, nocodazole, a microtubule assembly blocker, had no effects (*n*=9 cells, 10 μM). These results suggest that F-actin assembly pathways are involved in mediating Ω-profile merging. Interestingly, whole-cell dialysis of phalloidin–fluorescein isothiocyanate (FITC; 1.3 μM, 2-3 min), which stabilizes F-actin, inhibited Ω-shrink fusion, promoted stay fusion (Supplementary Fig. 3a,b), but did not affect the Lifeact intensity at the cell bottom (Supplementary Fig. 3c,d). These results suggest that dynamic F-actin polymerization is needed to mediate Ω-profile merging.

Consistent with dynamic F-actin assembly that requires ATP hydrolysis^17^, replacing ATP with adenosine 5-(γ-thio) triphosphate tetralithium salt (ATPγS) (2 mM, in the pipette solution), a non-hydrolysable ATP analogue, inhibited Ω-shrink fusion and increased stay fusion, but did not affect close fusion (Fig. 2b and Supplementary Fig. 4a). ATPγS also slowed down the *F*_647_ decay *τ* during Ω-shrink fusion (Fig. 2d and Supplementary Fig. 4b). Thus, ATP hydrolysis is needed to merge Ω-profiles.

Although the Ω-shrink fusion frequency is inversely related to ICa^11^, inhibition of Ω-shrink by actin-related drugs is not due to ICa changes for two reasons. First, these drugs did not affect ICa (Supplementary Fig. 5). Second, Lat A or Cyto D inhibited Ω-shrink and promoted stay fusion induced by whole-cell calcium dialysis, a stimulus independent of ICa (Supplementary Fig. 6).

**Figure 3:**
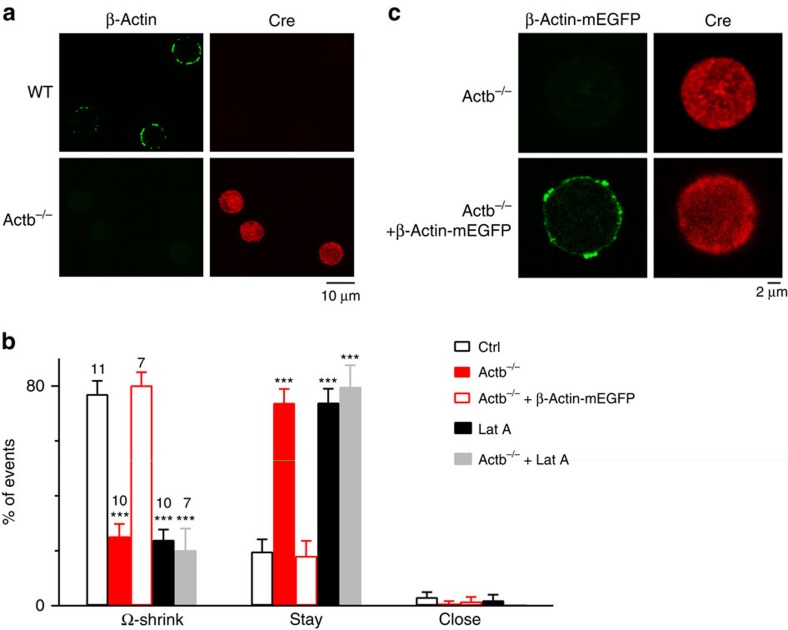
β-Actin KO inhibits Ω-shrink fusion but promotes stay fusion. (**a**) Immunostaining of β-actin in WT and Actb^−/−^ mouse chromaffin cells; Cre antibody staining was also shown to indicate cells expressed with Cre for deletion of *Actb1* gene. (**b**) Percentages (mean+s.e.m.) of Ω-shrink, stay and close fusion induced by whole-cell calcium (1.5 μM) dialysis in control (*n*=11 cells; total spot number: 96), Actb^−/−^ cells (*n*=10 cells; total spot number: 101), Actb^−/−^ cells overexpressed with β-Actin–mEGFP (*n*=7 cells; total spot number: 59), control cells treated with Lat A (*n*=10 cells; total spot number: 79 ) and Actb^−/−^ cells treated with Lat A (*n*=7 cells; total spot number: 63). ***P<0.001 (compared with control, Student's *t*-test). (**c**) β-Actin–mEGFP in Actb^−/−^ cells not transfected (upper) or transfected with β-Actin–mEGFP (lower). Cre antibody staining was also shown to indicate cells expressed with Cre.

To strengthen our conclusion, we studied how Ω-profile merging is affected when a major actin isoform, β-actin (encoded by *Actb* gene)^18^, is knocked out. KO was achieved by breeding chromaffin cell-specific Cre mice, the PNMT^Cre/+^ mice^19^, with Actb^LoxP/LoxP^ mice^20^ and subsequently breeding PNMT^Cre/+^; Actb^LoxP/+^ mice with PNMT^Cre/+^; Actb^LoxP/+^mice to generate PNMT^Cre/Cre^; Actb^LoxP/LoxP^ mice (Actb^−/−^ mice; Supplementary Fig. 7). Immunostaining of Cre and β-actin confirmed expression of Cre-recombinase and the absence of β-actin in Actb^−/−^ mouse chromaffin cells (Fig. 3a).

In cultured wild-type (WT) or Actb^LoxP/LoxP^ mouse chromaffin cells (control), Train_2Hz_ induced an ICa of 416±55 pA and a capacitance jump (*n*=10 cells; Supplementary Fig. 8a). Fusion events included Ω-shrink fusion (80±5%), stay fusion (18±5%) and close fusion (2±1%) as recorded at the confocal A647_strong_/A488_weak_ setting (Supplementary Fig. 8b–e; *n*=10 cells, total fusion spots: 80). Similar percentages of Ω-shrink, stay and close fusion were induced by whole-cell dialysis of calcium (1.5 μM) into the chromaffin cells (Fig. 3b). These results were similar to those observed in control bovine chromaffin cells. We used calcium dialysis as stimulation for most mouse experiments, because calcium dialysis induced more fusion events.

β-Actin KO reduced Ω-shrink from 77% in control to 25% and increased stay fusion from 20 to 74%, but did not affect close fusion during calcium dialysis (Fig. 3b). Similar reduction of Ω-shrink fusion and increase of stay fusion were obtained during Train_2Hz_ in Actb^−/−^ mouse chromaffin cells (Supplementary Fig. 8e). Rescue of KO by transfecting WT β-actin attached with monomeric enhanced green fluorescent protein (β-Actin–mEGFP, for recognition of transfected cells) to Actb^−/−^ cells increased β-actin expression (Fig. 3c; *n*=3 transfections) and rescued Ω-shrink fusion to the control level (Fig. 3b; *n*=7 cells). Thus, β-actin is involved in mediating Ω-profile merging. Application of Lat A (3 μM) in Actb^−/−^ cells did not further reduce Ω-shrink fusion (Fig. 3b; *n*=7 cells), but significantly reduced Ω-shrink fusion in WT cells (Fig. 3b; *n*=10 cells), confirming that Lat A reduces Ω-shrink fusion by blocking F-actin. These results consolidated our conclusion that actin is involved in mediating Ω-shrink fusion.

### F-actin is more concentrated near the plasma membrane

**Figure 4:**
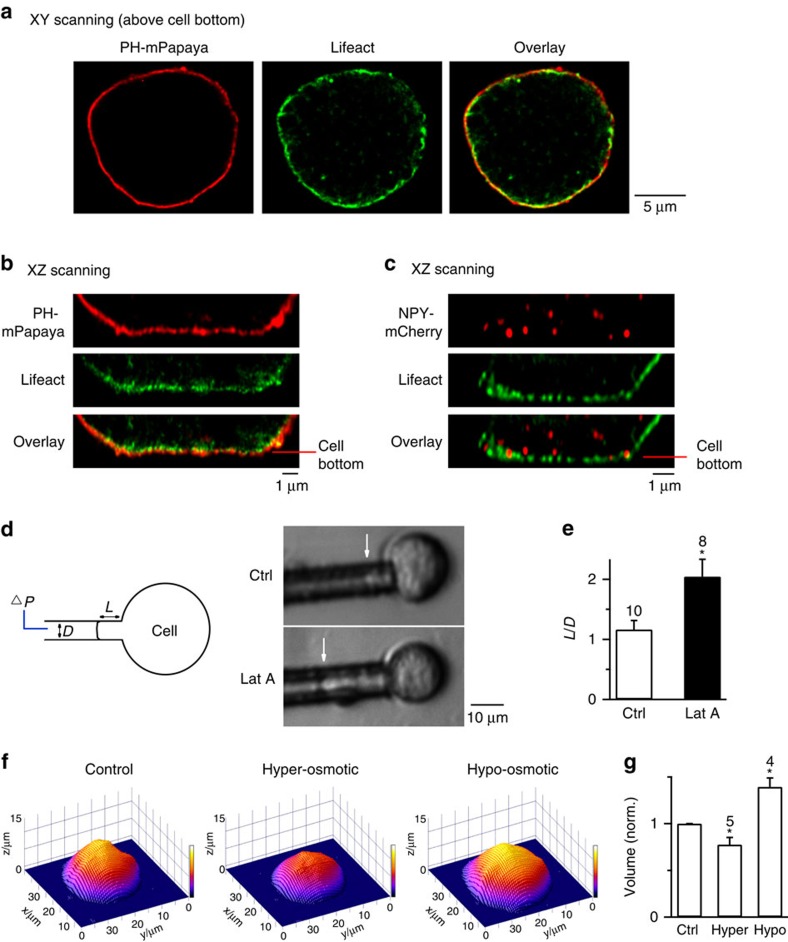
Localization of F-actin and manipulation of membrane tension by Lat A and by changes of osmolarity. (**a**) Sampled STED images of a cell overexpressed with PH–mPapaya (left, red, labelling the plasma membrane) and Lifeact-TagGFP2 (middle, green) at the conventional XY scanning mode with a focal plane >2 μm above the cell bottom. Left and middle panels are superimposed in the right panel. (**b**) STED XZ imaging of PH–mPapaya (upper) and Lifeact-TagGFP2 (middle) at the cell bottom. Upper and middle images are superimposed in the lower panel. (**c**) Sampled STED images of a cell overexpressing NPY–mCherry (upper, red) and Lifeact (middle, green) at the cell bottom, at the XZ scanning mode. Upper and middle panels are superimposed in the lower panel. (**d**) Lat A reduces cell surface tension. Left: drawings of micropipette aspiration technique. A negative pressure (Δ*P*) on the pipette (with a diameter *D*) draws the cell membrane into the pipette by a length *L*. Right: pipette-aspirated cells (bright-field images) in the absence (Ctrl) and presence of Lat A (0.5 μM). Arrows, membrane projection (*L*) in the micropipette (Δ*P*=500 Pa). (**e**) Normalized projection length (*L*/*D*, mean+s.e.m.) for aspirated cells in the absence (Ctrl, *n*=10 cells) or presence of Lat A (0.5 μM, *n*=8 cells; *P=0.011; unpaired two-tailed Student's *t*-test). Δ*P*=500 Pa. (**f**) Three-dimensional (3D) images of a chromaffin cell in control (Ctrl, 305 mOsm), in a hyper-osmotic solution (650 mOsm, Hyper) and a hypo-osmotic solution (164 mOsm, Hypo). The image was obtained with atomic force microscopy. (**g**) Chromaffin cell volume (mean+s.e.m.) in Ctrl, Hyper (*n*=5 cells, *P=0.03, paired two-tailed Student's *t*-test) or Hypo solution (*n*=4 cells, *P=0.016, paired two-tailed Student's *t*-test) computed from atomic force microscopic 3D images. Hyper and Hypo data were normalized to the Ctrl (305 mOsm).

Recent studies show that in cells containing ∼1–5 μm granules, F-actin coats the fusing Ω-profile with an onset at several seconds after fusion, which is thought to either compress or hold the Ω-profile^5^,^6^,^7^,^8^,^9^,^10^. Here we determined whether F-actin coats and shrinks the fusing Ω-profile in chromaffin cells. STED scanning at the microscopic XY plane above the cell bottom (> 2 μm) revealed that dense Lifeact co-localized with or near the overexpressed phospholipase C delta PH domain tagged with mPapaya (PH–mPapaya), which labels PtdIns(4,5)P_2_ (PIP_2_), a lipid localized at the plasma membrane (Fig. 4a)^21^. STED scanning at the microscopic *XZ* plane (vertical to *XY* plane, parallel to microscopic *Z* axis) at the cell bottom revealed that Lifeact closely adhered to the PH–mPapaya (Fig. 4b), confirming a dense cortical actin network observed near, but not far from, the plasma membrane (see also Supplementary Fig. 9a,b for quantification)^22^,^23^,^24^. With this dense actin network, granules near the plasma membrane, including NPY–mCherry-containing granules, were more associated with the F-actin network than those far from the plasma membrane (Fig. 4c), similar to a recent report with three-dimensional electron tomography^24^. We did not observe consistently actin rings surrounding NPY–EGFP-containing vesicles (Fig. 4c).

Confocal imaging revealed that in 41% (15 out of 37) of Ω-shrink fusion events, as *F*_647_ decayed due to Ω-profile shrinking, *F*_Lifeact_ increased at the spot centre (Supplementary Fig. 9c,d), suggesting F-actin movement or coating of the shrinking Ω-profile. However, the onset of *F*_Lifeact_ increase was 1.9±0.4 s (*n*=15) later than the onset of *F*_647_ decay that reflected Ω-profile shrinking (Supplementary Fig. 9c,d). *F*_Lifeact_ increase started when *F*_647_ decreased by 57±5% (*n*=15; Supplementary Fig. 9c,d). This delayed *F*_Lifeact_ increase suggests that F-actin movement or coating does not initiate Ω-profile shrinking. More importantly, 59% (22 out of 37) of Ω-shrink fusion events were not accompanied with *F*_Lifeact_ increase (Supplementary Fig. 9e,f). This lack of *F*_Lifeact_ increase was not due to the lack of Lifeact expression, because (1) Lifeact was expressed nearby Ω-shrink events showing no *F*_Lifeact_ increase (for example, Supplementary Fig. 9e) and (2) 15 out of 22 Ω-shrink events showing no *F*_Lifeact_ increase were observed in cells containing other Ω-shrink fusion events with *F*_Lifeact_ increase. Thus, Ω-profile shrinking takes place regardless of whether F-actin moves into the shrinking Ω-profile or not, suggesting that Ω-profile shrinking does not require F-actin recruitment to the shrinking Ω-profile.

We do not know why F-actin moves into the shrinking Ω-profile in some cases. It could be either a passive refilling of the empty space or an active process. Further understanding of this phenomenon is beyond the scope of the present work, which aimed at understanding the mechanisms underlying Ω-shrink fusion.

### F-actin exerts mechanical forces to merge the Ω-profile

F-actin increases the plasma membrane tension (Tension_pm_)^25^,^26^,^27^,^28^. Here we tested whether F-actin shrinks the Ω-profile via an increase of Tension_pm_. We estimated Tension_pm_, the force needed to pull the surface by certain length (force per unit length), using the micropipette aspiration technique. A negative pressure applied to a micropipette in contact with the plasma membrane drew the plasma membrane into the pipette to a length *L* (Fig. 4d)^29^. The normalized length, calculated as *L* divided by the pipette diameter *D* (*L*/*D*), is inversely correlated with Young's module, a membrane elasticity measurement that is correlated with Tension_pm_^29^. With a negative pressure of 500 Pa, the *L*/*D* value was ∼2 times larger in the presence of Lat A than in control (Fig. 4d,e), suggesting that Lat A reduces membrane stiffness and Tension_pm_ in chromaffin cells.

To determine whether reduction of Tension_pm_ inhibits Ω-shrink fusion, we changed Tension_pm_ with a commonly used method—increasing or reducing the bath solution osmolarity to reduce or increase Tension_pm_, respectively^25^,^28^. Consistent with reduced Tension_pm_, increasing osmolarity from 305 (control) to 650 mOsm (hyper-osmotic solution) reduced the cell volume, measured with atomic force microscopy (AFM; Fig. 4f,g). It also reduced the cell diameter, detected by confocal microscopy at the cell-centre with A647 in the bath (Supplementary Fig. 10a). Consistent with enhanced Tension_pm_^25^,^28^, reducing the bath osmolarity from 305 to 164 mOsm (hypo-osmotic solution) increased the cell volume (Fig. 4f,g) and diameter (Supplementary Fig. 10b), and increased Tension_pm_ estimated with micropipette aspiration (*n*=10 cells, not shown).

**Figure 5:**
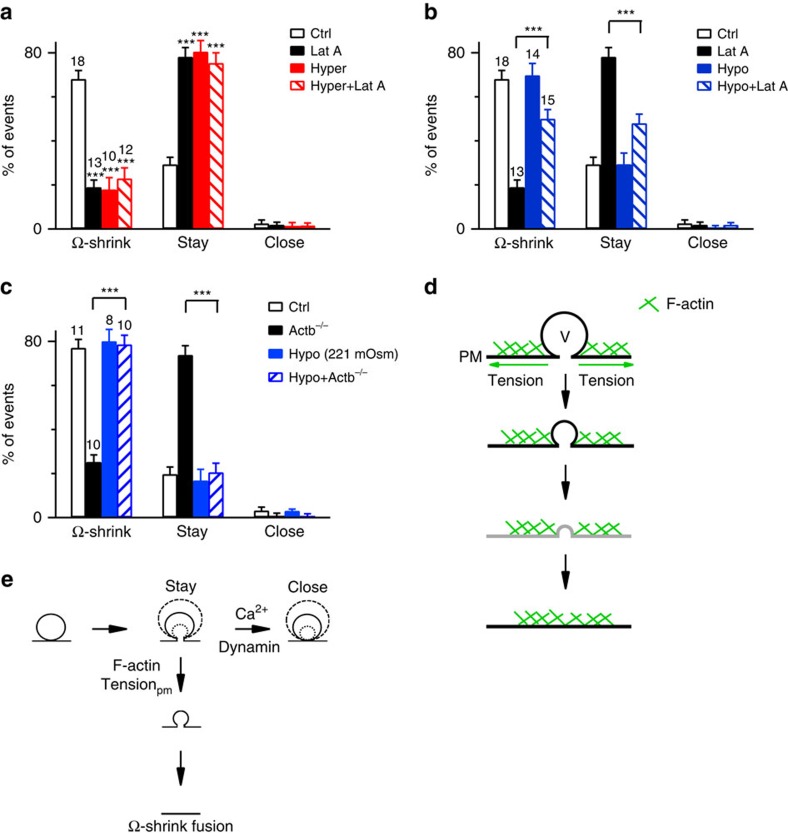
Actin provides sufficient Tension_pm_ to mediate Ω-profile shrinking. (**a**) Percentages (mean+s.e.m.) of Ω-shrink, stay and close fusion induced by Train_2Hz_ in Ctrl (305 mOsm, *n*=18 cells; 192 spots in total), in the presence of Lat A (3 μM, 305 mOsm, *n*=13 cells; 150 spots; ***P<0.001), in Hyper solution (*n*=10 cells; 70 spots, ***P<0.001) and in Hyper solution containing Lat A (*n*=12 cells; 100 spots, ***P<0.001) in bovine chromaffin cells. Statistical significances were assessed by unpaired two-tailed Student's *t*-test (applies to **a**–**c**). (**b**) Percentages (mean+s.e.m.) of Ω-shrink, stay and close fusion induced by Train_2Hz_ in Ctrl (305 mOsm, 18 cells, 192 spots), in the presence of Lat A (305 mOsm), in Hypo solution (*n*=14 cells, 220 spots; *P*=0.665) and in Hypo solution containing Lat A (*n*=15 cells, 276 spots, ***P<0.001) in bovine chromaffin cells. (**c**) Percentages (mean+s.e.m.) of Ω-shrink, stay and close fusion induced by whole-cell calcium (1.5 μM) dialysis in WT mouse chromaffin cells (WT, 305 mOsm, 11 cells, 96 spots), in Actb^−/−^ cells (305 mOsm, *n*=10 cells, 101 spots), in hypo-osmotic solution (221 mOsm) in WT cells (*n*=8 cells, 60 spots) and in the hypo-osmotic solution in Actb^−/−^ mouse chromaffin cells (*n*=10 cells, 82 spots, ***P<0.001). (**d**) A model showing that F-actin shrinks the Ω-profile by enhancing tension (T) at the plasma membrane (PM). Drawings in black are supported by our data. Drawings in grey are hypothetical (below our resolution limit). As the Ω-profile height equals its pore size, Ω-profile shrinking becomes Ω-profile collapse. (**e**) Updated Ω-exo-endocytosis model where F-actin provides Tension_pm_ to mediate Ω-shrink fusion and calcium triggers a dynamin-dependent close fusion (taken from ref. 11).

Resembling actin blockers, the hyper-osmotic solution decreased Ω-shrink fusion induced by Train_2Hz_ from 68 to 23% and increased the stay fusion from 29 to 75% (*P*<0.001; Fig. 5a). Adding Lat A to a hyper-osmotic solution did not further reduce Ω-shrink or increase the stay fusion percentage (Fig. 5a), suggesting that Lat A inhibited Ω-shrink fusion primarily by reducing Tension_pm_.

Compared with control, the hypo-osmotic solution did not further increase the Ω-shrink fusion or decrease stay fusion (Fig. 5b), probably because Ω-shrink fusion has already approached its maximal capacity (∼70%) in control. However, in the presence of Lat A, applying the hypo-osmotic solution increased Ω-shrink fusion from 19 to 50% (*P*<0.001; *t*-test), decreased stay fusion from 76 to 48% (*P*<0.001; *t*-test, 18 cells; Fig. 5b) and did not affect ICa (Supplementary Fig. 11). This rescue of Ω-shrink fusion by a large extent (Fig. 5b) suggests that the increase in Tension_pm_ by the hypo-osmotic solution counteracts Lat A's inhibitory effects on Ω-shrink fusion.

In summary, Lat A reduced Tension_pm_ (Fig. 4d,e) and Ω-shrink fusion percentage (Fig. 2b). Reducing Tension_pm_ by the hyper-osmotic solution mimicked Lat A's effect in inhibiting Ω-shrink fusion and prevented Lat A from further inhibiting Ω-shrink fusion (Fig. 5a). Conversely, enhancing Tension_pm_ by the hypo-osmotic solution largely prevented Lat A from inhibiting Ω-shrink fusion (Fig. 5b). These results suggest that Lat A inhibits Ω-shrink fusion by reducing Tension_pm_. In other words, F-actin mediates Ω-shrink fusion by providing sufficient Tension_pm_.

In Actb^−/−^ cells, applying the hypo-osmotic solution (221 mOsm) increased Ω-shrink fusion from 20 to 78% (*P*<0.001; *t*-test) and decreased stay fusion from 73 to 20% (*P*<0.001; *t*-test, *n*=10 cells; Fig. 5c). Applying the hypo-osmotic solution to WT mouse chromaffin cells did not increase the Ω-shrink fusion (Fig. 5c; *n*=8 cells). Thus, increasing Tension_pm_ by hypo-osmotic solution rescued the inhibition of Ω-shrink fusion by β-actin KO, which further supports our suggestion that actin mediates Ω-shrink fusion by providing sufficient Tension_pm_.

How would sufficient Tension_pm_ shrink the Ω-profile? Given that blocking F-actin increased stay fusion and thus the number of Ω-profiles at the plasma membrane, there should be an F-actin-independent force that maintains the Ω-profile from collapsing. With this Ω-profile-maintaining force, Tension_pm_ may pull the Ω-profile membrane and thus shrink the Ω-profile till merging of the Ω-profile with the plasma membrane (Fig. 5d). We can now update the Ω-exo-endocytosis model (Fig. 1c), where calcium and dynamin mediate close fusion^11^, with the involvement of F-actin and Tension_pm_ in Ω-shrink fusion (Fig. 5e).

### F-actin blockers increase Ω-profiles at active zones

**Figure 6:**
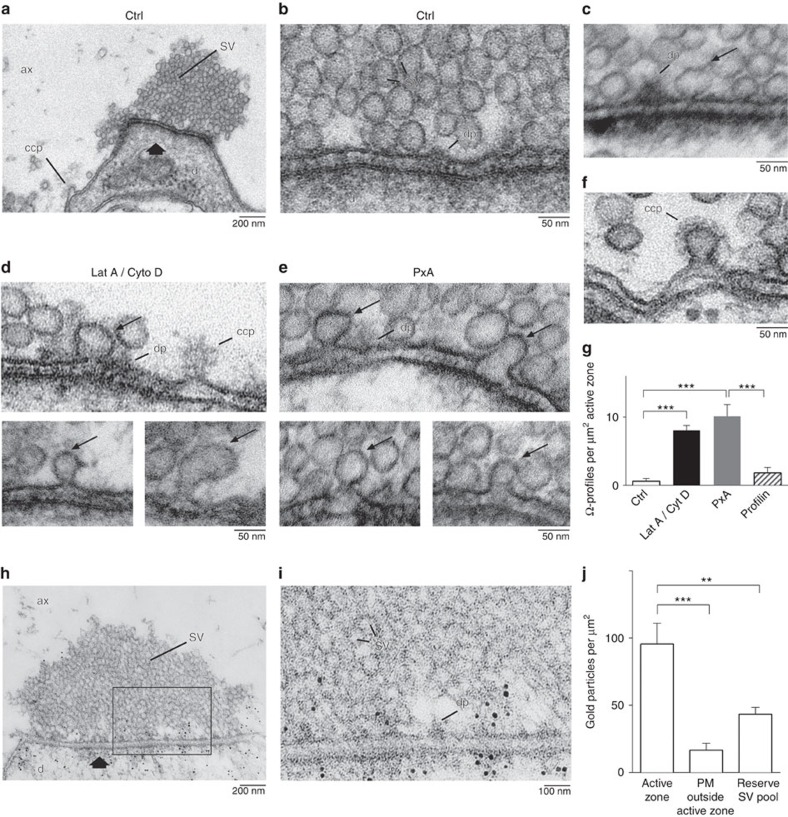
Actin-directed compounds induce Ω-profiles at lamprey active zones. (**a**) Electron micrograph of an active zone with a vesicle cluster (arrow) in a control reticulospinal synapse. The axon was stimulated at 5 Hz for 30 min. A clathrin-coated pit (ccp) is present lateral to the active zone. ax, axoplasmic matrix; d, dendrite; sv, synaptic vesicle. (**b**) An area of the active zone from **a** shown at higher magnification. dp, dense projection of the active zone. (**c**) An EM image showing a rare observation of an Ω-profile at the active zone membrane in the control active zone stimulated at 5 Hz for 30 min. (**d**,**e**) EM images of active zones in synapses microinjected with Lat A and Cyto D (**d**) or with PxA (**e**) and stimulated at 5 Hz for 30 min. Ω-profiles are marked with thin arrows. (**f**) An EM image showing clathrin-coated pits at the lateral region away from the active zone of a PxA microinjected synapse. (**g**) The number of Ω-profiles (mean+s.e.m.) at active zones in control (Ctrl) axons and in axons microinjected with Lat A (60 μM) and Cyto D (33 μM), with PxA (5 mg ml^−1^) or with profilin I (5 mg ml^−1^) (***P<0.001). Data are plotted as mean+s.e.m. per μm^2^ of active zone (AZ). Each group was from five synapses cut in serial section from three axons. Statistical significances were tested with one-way analysis of variance (ANOVA), α-level of 0.05, followed by the Holm–Šídák's test. ***P<0.001 for Ctrl versus Lat A/Cyto D, Ctrl versus PxA or Profilin versus PxA. *P*=0.43 for Ctrl versus Profilin. (**h**) Immunogold localization of actin immunoreactivity at active zones. A synapse fixed at rest showing gold particles near the presynaptic active zone and the postsynaptic density. (**i**) The box region in **h** shown at higher magnification. (**j**) Bar graph showing the number of actin gold particles per μm^2^ within 100 nm from the active zone membrane, within 100 nm from the presynaptic membrane, but ∼300 nm away from the active zone (PM outside active zone, see Supplementary Fig. 13a for an illustration of these regions), and in the reserve pool area 200 nm from the active zone (*n*=20 synapses, mean+s.e.m.; **P<0.01; ***P<0.001, Student's *t*-test).

To determine whether F-actin mediates Ω-profile merging in synapses, we examined whether block of actin dynamics accumulates Ω-profiles in lamprey giant reticulospinal synapses, in which intermediates of the vesicle cycle can be clearly resolved using EM^30^,^31^. We microinjected giant axons with a mixture of Lat A and Cyto D or a modified profilin–actin protein with a zero-length covalent cross-link between profilin and actin, termed PxA^32^,^33^,^34^. PxA structually resembles endogeneous profilin–actin, but is non-dissociable between profilin and actin, and thus inhibits formin-dependent F-actin assembly^32^,^33^,^34^. Concentrations of the actin-directed compounds in microinjection pipette were 60 μM per 33 μM for Lat A/Cyto D and 5 mg ml^−1^ for PxA, but were diluted ∼10–20 times in the studied regions of axons following microinjections (Supplementary Fig. 12a). The active zone morphology (Supplementary Fig. 12b–e and Fig. 6a,b) and the action potential progagation along the axon (Supplementary Fig. 12f) were apparently normal after microinjection. Synapses were stimulated with action potentials at 5 Hz for 20–30 min, then fixed and embedded for EM in serial ultrathin sections^30^,^31^.

Vesicle clusters were observed at active zones and examined in serial ultrathin sections (Fig. 6a–f and Supplementary Fig. 12b–d). Ω-profiles were identified as having a pore smaller than or equal to its width (Fig. 6c-e). Ω-profiles were rarely observed in control synapses or synapses microinjected with bovine profilin I (Fig. 6c,g), but were much more frequently observed at the active zones of the synapses microinjected with Lat A/Cyto D (Fig. 6d,g) or PxA (Fig. 6e,g). Lat A/Cyto D or PxA did not significantly affect the number of vesicles at the active zone membrane (Supplementary Fig. 12b–e). Unlike clathrin-coated Ω-profiles observed at the periactive zone (Fig. 6f)^30^,^31^, Ω-profiles at the active zone were not coated with the clathrin lattice (Fig. 6c-e), suggesting that they are not generated via clathrin-mediated endocytosis. Dimension of most of Ω-profiles were similar to vesicles, but some appeared to be larger or smaller (for example, Fig. 6d,e and Supplementary Fig. 12b–d), consistent with stay fusion displaying variations of Ω-profile size in chromafin cells (Supplementary Fig. 1)^11^.

The Ω-profiles accumulated at the active zone are unlikely to be due to block of the fusion pore closure, because in chromaffin cells actin blockers did not block close fusion induced by Train_2Hz_ (Fig. 2) or a 1 s depolarization (*n*=8 cells, 3 μM Lat A, not shown), the latter of which induced more close fusion events^11^. Analogous to results observed in chromaffin cells, results observed in lamprey axons (Fig. 6) suggest that F-actin promotes Ω-profile merging at active zones. This suggestion was further supported by the following two sets of control experiments.

First, post-embedding immunoEM with a monoclonal actin antibody^12^,^19^ revealed an accumulation of immunogold particles around dense projections of the active zone in giant synapses (Fig. 6h–j and Supplementary Fig. 13a–d)^35^. The number of gold particles near the presynaptic plasma membrane (<100 nm) of the active zone (95.7±15.4 μm^−2^; *n*=20 synapses) was significantly higher than that (the particles near the presynaptic plasma membrane) >300 nm outside the active zone (PM outside active zone, 16.9±5.0 μm^−2^; *n*=20 synapses, *P*<0.001; Fig. 6j and also see Supplementary Fig. 13a–d for illustrations). It was also significantly higher than in the synaptic vesicle reserve pool area, 200 nm inside the active zone membrane (43.5±5.0 μm^−2^; *n*=17 synapses, *P*=0.0048; Fig. 6h–j). Thus, actin network is denser near the active zone membrane. The dense actin network at the active zone membrane was further confirmed by confocal microscopy of F-actin labelled with phalloidin conjugated with Alexa Flour 488 (phalloidin-Alexa 488) injected into the axon (Supplementary Fig. 14a). Microinjected phalloidin-Alexa 488 accumulated in spots of fluorescence that labelled active and periactive zones^36^ (see also Supplementary Fig. 14a).

Second, to determine whether Lat A and Cyto D reduced F-actin, we injected Lat A/Cyto D plus Texas Red and 10–15 min later injected phalloidin-Alexa 488 into the axon at ∼800 μm away. Next, we measured phalloidin-Alexa 488 fluorescence punta at ∼300–500 μm away from the Lat A/Cyto D injection site (Supplementary Fig. 14b,c). The number of detectable puncta was decreased by five- to tenfold in the area where compounds intermixed and the average phalloidin-Alexa 488 punta intenstiy was significantly lower in Lat A/Cyto D-injected axons (43.1±5.1 arbitrary units, *n*=15 spots, 2 axons; Supplementary Fig. 14c,d) than in control axons injected with Texas Red only (72.8±2.0 arbitrary units, *n*=29 spots, 2 axons; Supplementary Fig. 14b,d; *P*<0.0001, two-tailed Student's *t*-test), suggesting that Lat A and Cyto D reduced F-actin in lamprey axons.

In summary, actin was localized in the active zone membrane (Fig. 6h–j and Supplementary Figs 13 and 14). F-actin directed compounds reduced F-actin (Supplementary Fig. 14) in the axon and caused accumulation of Ω-profiles at the active zone membrane (Fig. 6) without damaging the morphology and the action potential propagation of the axon (Supplementary Figs 12 and 13). These results suggest that F-actin is involved in mediating Ω-profile merging at the neuronal nerve terminal.

## Discussion

The present work establishes a molecular and biophysical model accounting for Ω-profile merging, which defines the modes of exocytosis and endocytosis. We found that dynamic F-actin assembly is necessary for Ω-profile merging in chromaffin cells (Figs 1, 2, 3). F-actin imaging and Tension_pm_ measurements and manipulations suggest that F-actin provides sufficient Tension_pm_ to facilitate Ω-profile shrinking and thus to merge the Ω-profile with the plasma membrane (Figs 4 and 5). Not only F-actin but also the F-actin assembly pathway such as N-WASP, formin (Figs 2 and 6) and ATP hydrolysis (Fig. 2) may participate in Ω-profile merging. The highly abundant cytoskeletal actin and its assembly pathway, the essential energy-providing molecule ATP and cell membrane tension controlled by many factors may thus regulate exo- and endocytosis in physiological and pathological conditions by controlling Ω-profile merging.

Our findings, obtained from bovine and mouse chromaffin cells containing large vesicles, may apply to many other cells containing similarly large dense-core vesicles. They may apply to large vesicles distributed widely in glial cells, nerve terminals, neuronal dendrites and cell bodies that secrete dopamine, peptides or hormones (for example, oxytocin and vasopressin) or deliver transmembrane receptors to dendrites and dendritic spines crucial for our brain's physiological functions and disorders^2^,^37^.

Our finding that F-actin mediates Ω-profile merging is likely to apply to small synaptic vesicles, because (1) actin blockers accumulate Ω-profiles at lamprey active zones (Fig. 6) and (2) neuroendocrine chromaffin cells and nerve terminals are similar in the molecular machineries of exo- and endocytosis^1^,^2^,^38^,^39^,^40^. We acknowledge that the evidence obtained with EM at lamprey synapses was not as direct as that in live chromaffin cells where Ω-profile merging could be observed directly. The final resolution at live synapses, although technically not available now, will eventually be needed to establish our conclusion for synapses.

Ω-profile merging is via shrinking of Ω-profile in chromaffin cells^11^, but is generally believed to be via pore dilation (full-collapse fusion) at synapses^41^,^42^,^43^. One may wonder how F-actin mediates these two different behaviours. Ω-shrink fusion was revealed in live cells with STED imaging at ∼90 nm resolution, which could not exclude pore dilation below 90 nm^11^, whereas pore dilation was suggested based on EM^41^,^42^,^43^ but has not been visualized and proved in any live cell. Ω-profile shrinking and pore dilation may therefore be reconciled with a scenario, in which Ω-profile membrane was pulled into the plasma membrane by Tension_pm_, resulting in continuous Ω-profile shrinking until the height of the Ω-profile is shorter than the pore diameter (Fig. 5d, grey drawing). Under this condition, further pulling of the Ω-profile membrane into the plasma membrane by Tension_pm_ results in a collapse-like structure, turning into a full-collapse fusion (Fig. 5d). This model requires an actin-independent mechanism to hold the pore from collapse, which must exist because F-actin inhibition increased stay fusion, the persistent presence of fusion-generated Ω-profiles (Figs 2 and 3). The pore size may determine when the merging process changes from shrinking to collapse. It would be of great interest to test this model by visualizing the fusion pore during shrinking of the Ω-profile in the future.

Although inhibition of F-actin reduced Ω-shrink, but increased stay fusion, close fusion did not increase (Figs 2 and 3). This observation suggests that conversion from stay to Ω-profile closure is not automatic but requires a trigger. We recently found that calcium influx triggered close fusion—large calcium influx during a 1 s depolarization induce close fusion in > 80% of fusion events and nearly no Ω-shrink fusion^11^. Thus, large calcium influx is needed to convert stay to close fusion. This may explain why with relatively low calcium influx during Train_2Hz_, increase of stay fusion in the presence of F-actin blockers did not significantly increase close fusion. Taken together, the extent of calcium influx determines whether the fusing Ω-profile closes or not (Fig. 5e). If the Ω-profile does not close, sufficient Tension_pm_ provided by F-actin may mediate Ω-shrink fusion (Fig. 5e). If Tension_pm_ is insufficient, Ω-profile stays at the plasma membrane, resulting in the stay fusion (Fig. 5e).

With large calcium influx, close fusion occurs much more often than Ω-shrink fusion^11^. This might be in part due to inhibition of Ω-shrink fusion by strong calcium influx. Consistent with this possibility, calcium influx facilitates F-actin disassembly^44^, which may result in reduction of Ω-shrink fusion (Fig. 2). It would be of great interest to prove (or disapprove) this possibility with experimental evidence in the future.

A large number of previous studies suggest that actin may play several possibly overlapping roles in the fusion process, including serving as a fusion barrier or providing a scaffold for anchoring secretory vesicles, a track to deliver secretory vesicles to the fusion site, regulation of vesicle docking and priming, regulation of the fusion pore, regulation of membrane merging after fusion and endocytosis (reviewed in ref. 45). As discussed below, the present work suggests a new mechanism of F-actin—mediating Ω-profile shrinking and merging by providing sufficient Tension_pm_.

In *Xenopus* oocytes containing cortical granules^5^, human endothelial cells containing Weibel–Palade bodies, lacrimal epithelial acinar cells containing zymogen granules^6^, parotid or pancreatic acinar cells containing zymogen granules^7^,^8^ and type II pneumocytes containing lamellar body^9^, vesicle fusion is followed by F-actin coating of the fusing vesicle (reviewed in ref. 10). Such an F-actin coating has been suggested to mediate two seemingly opposite functions: to compress the fusing Ω-profile and thus to expel the vesicular content, or to hold the Ω-profile from merging into the plasma membrane. However, these results were obtained from vesicles (∼1–5 μm in diameter), which are much larger than those in neuroendocrine chromaffin cells (∼300 nm) and neurons (∼20–80 nm), and take much longer time, in the order of tens of seconds to tens of minutes, to merge the Ω-profile and expel vesicular contents. These substantial differences might explain why F-actin recruitment to the fusing vesicle is not the main mechanism to shrink the Ω-profile in chromaffin cells (Supplementary Fig. 9). Instead, F-actin provides sufficient Tension_pm_ (Figs 4 and 5) to mediate rapid Ω-profile merging in hundreds of milliseconds in chromaffin cells (Figs 2 and 3). It is possible that this mechanism may also contribute to mediate Ω-profile merging in cells containing large 1–5 μm vesicles, given that Tension_pm_ has not been considered for large vesicle fusion in previous studies.

F-actin blockers slow down catecholamine release^46^,^47^,^48^ and NPY–EGFP release (Supplementary Fig. 15) in chromaffin cells, suggesting that F-actin speeds up vesicular content release^49^. It was suggested that F-actin may squeeze the fusing Ω-profile to speed up vesicular content release^49^. Our results suggest that the force to expel vesicular contents may be from Tension_pm_ provided by F-actin, but not from active F-actin coating of the fusing Ω-profile as observed during extremely slow release from very large vesicles^10^. In addition to facilitating content release, F-actin may promote dispersion of vesicular membrane proteins by promoting Ω-profile shrinking, because a stabilized Ω-profile retains vesicular membrane protein VAMP2 (ref. 11).

A previous study observed that actin-directed compounds increase the quantal size (from amperometric measurements) and reduce the capacitance variance^48^,^50^ in chromaffin cells. This observation led to the suggestion that actin stabilizes Ω-profiles in chromaffin cells containing ∼300 nm vesicles^50^. However, this suggestion is interpreted from the measurements of quantal size (from amperometric measurements) and capacitance variance, which could not estimate a pore beyond ∼5 nm^51^,^52^ nor predict Ω-profile merging. By direct measurements of Ω-profile merging, we found that F-actin mediates Ω-profile merging. We therefore suggest not to interpret Ω-profile merging based on indirect amperometric or capacitance measurements.

Although fusion pore opening is well known to overcome a significant energy barrier^4^, once the pore is opened, the Ω-profile is generally considered an unstable fusion intermediate that rapidly and passively merges with the plasma membrane without consuming energy in endocrine cells and neurons. This view appears to be incorrect, as we found that ATP hydrolysis and dynamic actin polymerization are needed to merge the Ω-profile. The fusion-generated Ω-profile is actually a stable structure in the absence of ATP hydrolysis (Fig. 2). Given that Ω-profile merging takes place in all secretory cells^1^,^2^, Ω-profile merging might be an energy-consuming step previously unrecognized. As vesicle exo- and endocytosis consume most ATP molecules in nerve terminals^53^, ATP consumption in Ω-profile merging is a factor worth of consideration in low metabolic states with low ATP levels, mitochondria dysfunctions in various diseases (for example, Parkinson's disease and Alzheimer's disease) that reduce ATP^54^ and in diseases that affect F-actin or F-actin assembly pathways^55^.

## Methods

### Primary bovine chromaffin cell culture

Bovine chromaffin cells were prepared as described previously^11^. Briefly, fresh adrenal glands were obtained from a local slaughterhouse on the day of culture. After excess fat was trimmed off the glands (two were used per culture), glands were then perfused with cold 1 × Lock's buffer containing (in mM): NaCl, 145; KCl, 5.4; Na_2_HPO_4_, 2.2; NaH_2_PO_4_, 0.9; glucose, 5.6; and HEPES, 10 pH 7.3, to remove any residual blood. Each gland was then injected through the portal vein with ∼2 ml of filtered Lock's buffer containing collagenase P (1.5 mg ml^−1^, Roche), trypsin inhibitor (0.325 mg ml^−1^, Sigma) and BSA (5 mg ml^−1^, Sigma), and incubated at 37 **°**C for 20 min in a water bath. The glands were then cut open longitudinally, to expose the digested medulla. The medulla were carefully removed, minced in Lock's buffer and filtered through a nylon mesh. The filtrate was centrifuged at 500 r.p.m. for 4–5 min, to obtain the cell pellet. The supernatant was then removed and the pellets resuspended in Lock's buffer. The process was repeated one to two times until the supernatant was clear. Final cell pellet was resuspended in pre-warmed DMEM low glucose medium (Gibco) supplemented with 10% fetal bovine serum (Gibco) and plated onto poly-D-lysine and laminin I-coated 25 mm-diameter glass coverslips (Neuvitro Corp., USA). The plated cells were incubated at 37 **°**C, 8% humidified CO_2_ and used within 4 days after culturing. In some experiments, cells were transfected with 2 μg of Lifeact-tagGFP2 (Ibidi, Germany) by electroporation using a basic neuron nucleofector kit (Lonza, Program O-005) according to the manufacturer's instruction.

### Electrophysiology

Whole-cell voltage-clamp and capacitance recordings were performed with an EPC-10 amplifier together with the lock-in software (PULSE, HEKA, Lambrecht, Germany)^56^. All experiments were carried out at room temperature (21 °C −24 °C) with the cells immersed in bathing solution containing (in mM): 125 NaCl, 10 glucose, 10 HEPES, 5 CaCl_2_, 1 MgCl_2_, 4.5 KCl, 0.001 tetrodotoxin and 20 tetraethylammonium ion, pH 7.3. The osmolarity of the bath solution was 305–310 mOsm. In some experiments, we increased the osmolarity to 640–650 mOsm by adding 295 mM sucrose to the bath solution. In some experiments, we decreased the osmolarity to 160–221 mOsm by reducing NaCl to 55–70 mM.

The pipette (3–6 MΩ) solution contained (in mM): 130 glutamate, 0.5 EGTA, 12 NaCl, 30 HEPES, 1 MgCl_2_, 2 ATP and 0.5 GTP pH 7.2 adjusted with CsOH. The osmolarity was ∼308 mOsm. In some experiments, ATP (2 mM) in the pipette solution was replaced with a non-hydrolysable ATPγS (2 mM, Sigma) and MgCl_2_ was raised to 3 mM to facilitate endogenous ATP turnover^57^. For intracellular Ca^2+^ dialysis experiments, the pipette solution contained (in mM): 110 glutamate, 10 EGTA, 12 NaCl, 30 HEPES, 1 MgCl_2_, 2 ATP 0.5 GTP and 9 CaCl_2_ pH 7.2 adjusted with CsOH. The free Ca^2+^ concentration was ∼1.5 μM, which was calculated based on the Max-Chelator programme (Stanford University, Stanford, CA), in which the calcium dissociation constant of EGTA is 0.15 μM^58^,^59^.

The holding potential was −80 mV. For Train_2Hz_ stimulation, a Train of 50 ms depolarization from −80 to +10 mV was given at 2 Hz; each 50 ms depolarization was preceded by a 50 ms pre-pulse from −80 to +120 mV, to facilitate Ca^2+^ channel current^60^,^61^,^62^. Cell membrane capacitance was simultaneously recorded 15 s before, during and 30 s after depolarization. The sample interval for current recordings was 50 μs. The frequency of the sinusoidal stimulus was 1,000–1,500 Hz with a peak-to-peak voltage ≤50 mV.

### Confocal imaging

Alexa 647 (A647; 20–30 μM in bathing solution, Invitrogen) and Alexa 488 dyes (A488; 20–30 μM in bathing solution, Invitrogen) were excited by a solid-state 638 nm (30 mW output) and 488 nm lasers (20 mW output) with an inverted confocal microscope (TCS SP8, Leica, Germany; original magnification, × 63/1.40 oil objective). Unless mentioned otherwise, the 638 nm laser was set at 20–25% of the maximum power and the 488 nm laser was set at 0.5–2% of the maximum power. A647 fluorescence was collected with a photomultiplier at 639–767 nm, whereas A488 was collected with a hybrid GaAsP spectral detector at 489–596 nm. For time-lapse A647/A488 imaging, images were collected with 40 ms inter-frame interval at 45–70 nm per pixel in an imaging area of ∼160–320 μm^2^. In some experiments, cells were pre-treated with 3 μM latrunculin A (Enzo Life Sciences), 4 μM Cyto D (Enzo Life Sciences), wiskostatin (10 μM, Tocris Bioscience) and SMIFH2 (25 μM, Tocris Bioscience) for 20 min in the bath solution, or whole-cell dialysis of phalloidin–FITC (1.3 μM, Invitrogen) for 2–3 min before imaging.

### STED imaging

The inverted STED microscopes used in this study (TCS SP5 STED, TCS SP8 STED 3X, Leica) have a resolution of ∼60–90 nm. A488 (60 μM) was excited with an Argon laser at 488 nm at 20% of the maximum power (maximum power: 25 mW) and depleted with a continuous wave fibre laser at 592 nm using the maximum power (1.5 W). The fluorescence was acquired by GaAsP hybrid detection system at 498–580 nm. At 20% of the maximum power, 488 nm laser caused A488 bleaching after pore closure with a time course similar to that of A647 under the confocal setting^11^. Lifeact (TagGFP2) was excited by a tunable white light laser at 470 nm (7% of maximum power) with the STED depletion laser at 592 nm (25% of the maximum power) and its fluorescence at 480–560 nm was collected using time-gated detection (1.5–6.5 ns). mCherry was excited by the tunable white light laser at 570 nm (6% of maximum power) with the STED depletion laser at 660 nm (60% of the maximum power) and its fluorescence between 575 and 650 nm was collected using time-gated detection (0.5–6.5 ns). PH–mPapaya was excited by the tunable white light laser at 530 nm (14% of the maximum power) with the STED depletion laser at 660 nm (10% of the maximum power) and its fluorescence between 541 and 620 nm was collected using time-gated detection (1.5–6.5 ns). When NPY–mCherry or PH–mPapaya and Lifeact (TagGFP2) were imaged at the STED microscope, NPY–mCherry (or PH–mPapaya) was imaged first, to avoid bleaching of mCherry or mPapaya fluorescence by the 592 nm STED depletion laser. For XZ scanning, we used 70–80% of STED depletion laser power in the *Z*-direction to improve the *z* axis resolution.

### Image analysis

A647 or A488 spots (Ω-profiles filled with A647 or A488) were identified during and <3 s after the end of depolarization. The spot fluorescence intensity was measured using the LAS AF Life analysis tool or Image J and plotted using Igor Pro 6.12. The baseline fluorescence value for each spot was normalized to 1. The representative image frames shown below the fluorescence traces were either unaveraged or averaged up to 20 frames as indicated in the figure legends. *W*_H_ was measured from intensity profiles of two to four lines across the spot centre at 45^o^ or 90^o^ apart^11^.

Although fusion modes (Ω-shrink fusion, stay and close fusion) were identified mostly at the cell-bottom confocal A647/A488 setting (strong/weak excitation), we sometimes identified the fusion modes with a single dye excited strongly, as described in detail in ref. 11. A single dye was used for Actb^−/−^ cells overexpressed with β-Actin–mEGFP (Fig. 3b), for cells dialysed with phalloidin–FITC (Supplementary Fig. 3) and for STED imaging above the cell bottom (Supplementary Fig. 1c; 592 nm laser for depletion).

### Generation of chromaffin cell-specific β-actin KO mice

Global KO of β-actin is embryonically lethal^63^,^64^. Accordingly, we used a chromaffin cell-specific Cre mouse, the PNMT^Cre/+^ mouse^19^, to knock out β-actin tissue specifically in chromaffin cells. We bred the 129/SvImJ PNMT^Cre/+^ mice with C57BL/6 Actb^LoxPLloxP^ mice (*Actb* gene encodes β-actin)^20^ and subsequently bred PNMT^Cre/+^; Actb^LoxP/+^ mice with PNMT^Cre/+^; Actb^LoxP/+^ mice to generate PNMT^Cre/Cre^; Actb^LoxP/LoxP^ mice (Actb^−/−^ mice). Mouse genotypes were determined by PCR.

### Mouse chromaffin cell culture

Animal care and use were carried out in accordance with NIH guidelines and approved by NINDS/NIDCD/NCCIH Animal Care and Use Committee in NIH. Five to 10 weeks old female or male mouse adrenal glands were used for experiments to obtain chromaffin cells as described previously^65^ with some modification. Briefly, adrenal glands were removed from mice (two per culture) and bathed in a dissociation buffer containing (in mM): 80 Na-glutamate, 55 NaCl, 6 KCl, 1 MgCl_2_, 10 HEPES and 10 D-glucose pH 7 adjusted with NaOH. Under a dissecting microscope, adrenal cortex was carefully removed to expose the medulla. The medulla was first digested in the dissociation buffering containing papain (30 U ml^−1^, Sigma), BSA (0.5 mg ml^−1^, Sigma) and dithiothreitol (0.1 mM, Sigma) at 37 °C for 8 min and further digested in the dissociation buffer containing collagenase F (3 U ml^−1^, Sigma) and CaCl_2_ (0.1 mM) at 37 °C. The digested medulla was carefully resuspended in DMEM medium (Invitrogen) containing 10% fetal bovine serum (Gibco) and centrifuged at 500 r.p.m. for 3 min. For rescue experiments, Actb^−/−^ cell pellet was transfected with β-Actin–mEGFP (2 μg, Addgene) using a basic neuron nucleofector kit (Lonza, Program O-005). For most other experiments, cell pellet was resuspended in the fresh pre-warmed DMEM and plated onto poly-D-lysine and laminin I-coated 25 mm-diameter glass coverslips (Neuvitro Corp.). Cells were incubated at 37 °C with 8% CO_2_ for at least 48 h before using for imaging or immunocytochemistry. For immunocytochemistry, cultured cells were briefly washed with 1 × PBS and fixed with 4% paraformaldehyde (Electron Microscopy Sciences, USA) in PBS for 20 min. Cells were permeabilized with 0.5% Triton X-100 for 10 min, blocked with 5% goat serum before incubation with mouse anti-β-actin (1/500, Abcam) and rabbit anti-Cre-recombinase antibodies (1/250, Abcam) in PBS containing 0.1% Triton X-100 and 1% BSA at 4 °C overnight. After washing with PBS, cells were incubated with Atto-488- and Alexa-647-conjugated secondary antibodies (1/400) in PBS containing 1% BSA, washed with PBS and mounted. Images were examined by Zeiss LSM 510 meta confocal microscope. Same image settings were applied to both control (WT or Actb^LoxP/LoxP^) and β-actin KO mice chromaffin cells.

### Micropipette aspiration measurements

A home-built micropipette aspiration system with a graduated manometer was used for application of controlled suction pressure onto living cells. Glass micropipettes with an inner diameter of 2–3 μm were filled with 0.2% BSA in 1 × PBS, to allow smooth movement of the cell membrane inside the pipette. Negative pressure in the micropipette tip was generated by aspirating water from the manometer reservoir and increased gradually in 200 Pa increments. Nikon Advanced Modulation Contrast optics mounted on a Nikon Ti-S microscope and CoolSnap MYOCCD camera (Photometrics, Tucson, AZ) were used to acquire live-cell bright-field images. Image J (http://rsb.info.nih.gov/ij/) software was used to manually measure the pipette diameters and plasma membrane projection length within the pipette.

### Atomic force microscopy

AFM imaging was performed on a BioScope Catalyst II AFM with a Nanoscope V controller (Bruker, CA). A polystyrene microsphere with known diameter of 9.6 μm (Interfacial Dynamics Corp., Portland, OR, USA) was glued onto a tipless cantilever (MLCTO10-D, Bruker) whose spring constant is calibrated by the standard thermal fluctuation method. The cell volume was obtained through the force volume mode in a scan range of 40 × 40 μm with 64 × 64 pixels. The volume of the same cell was measured in control, in a hyper-osmotic solution and in a hypo-osmotic solution under the same probe and parameter setting for direct comparison. The resulting topographic images were exported to ImageJ (version 1.49d, NIH, Bethesda, MD) for three-dimensional visualization and cell-shape computation, and for final volume ratio statistics and plotting in Microsoft Excel (version 14, Richmond, WA).

### Data collection and statistical analysis in chromaffin cells

All data at the whole-cell configuration were collected within 3 min after whole-cell break-in, which minimized whole-cell rundown of endocytosis^11^,^66^. All experiments in chromaffin cells were carried out from at least three independent cultures. Unpaired or paired Student's *t*-test (two-tailed) was used for the statistical analysis. The data were expressed as mean±s.e.m. or mean+s.e.m.

### Lamprey synapse and EM

Animal care and use were carried out in accordance with Karolinska Institutet guidelines and approved by the Swedish Board of Agriculture. Dissection of the trunk region of the lamprey spinal cord, microinjection procedures, stimulation and fixation were performed as described before^67^. Giant axons were stimulated at 5 Hz for 20–30 min using an extracellular electrode. Action potentials in giant axons were monitored with an extracellular suction electrode placed at the caudal part of the preparation (Supplementary Fig. 12f). The microinjection pipettes contained latrunculin A (60 μM) and Cyto D (33 μM), phalloidin-Alexa Flour 488 (1,200 U μl^−1^; Invitrogen), PxA (5 mg ml^−1^) and bovine profilin I (5 mg ml^−1^) in a buffer solution containing 10 mM HEPES (pH 7.4), 250 mM K^+^ acetate and 0.5% dimethyl sulfoxide. Covalently cross-linked PxA was prepared as described previously^33^. Compounds were diluted in Texas Red (3,000 Mw, Molecular Probes) and monitored with a charge-coupled device camera (Princeton Instruments) connected to a fluorescence microscope and a Nikon D-eclipse C1 confocal microscope using a × 10 air or × 40 water-immersion objective (numerical aperture 0.80).

Synapses were studied for EM at distances larger than 100 μm from the site of microinjections where the Texas Red fluorescence was 10–20 times lower than at the site of microinjection (Supplementary Fig. 12a). We therefore estimated that the concentration of Lat A and Cyto D at the site of EM examination was <6 and 3.3 μM, respectively.

Spinal cords were fixed during stimulation with 3% glutaraldehyde, 0.5% paraformaldehyde in 0.1 M cacodylate buffer (pH 7.4) containing 4% tannic acid for 1 h, followed by the same fixative without tannic acid for 3 h. After post fixation in 1% osmium tetroxide for 1 h and dehydration in alcohol, the specimens were embedded in Durcupan ACM (Fluka). Serial ultrathin sections were cut with a diamond knife (Diatome) and viewed at 80 kV in a Tecnai 12 electron microscope (FEI).

Effects of microinjections were analyzed in synapses cut in serial ultrathin sections. Samples were blinded before imaging and analysis. All described effects were reproduced in at least two independent preparations in which one to six axons were microinjected. Quantifications of fusion intermediates and synaptic vesicles at the active zone were done from serial sections of five synapses. Significances and *P*-values were calculated using one-way analysis of variance followed by Holm–Šídák's multiple comparisons test (GraphPad Prism 6).

For immunogold labelling experiments, non-stimulated lamprey spinal cords were dissected, fixed in 3% paraformaldehyde and 0.5% glutaraldehyde in 0.1 M cacodylate buffer (pH 7.4) containing 4% tannic acid for 1 h at 4 °C, followed by the same fixative without tannic acid for 3 h. Specimens were stained *en bloc* with 2% uranyl acetate, dehydrated in graded ethanol series and embedded at −25 °C in LR Gold resin (Fluka). Serial ultrathin sections were collected on nickel mesh grids and incubated overnight at 4 °C with primary monoclonal actin antibodies (β-actin Antibody (C4): sc-47778; Santa Cruz Biotech) diluted 1:100 in Tris-PBS (pH 7.4) containing 1% human serum albumin and 5% BSA. Secondary antibodies conjugated to 5 nm colloidal gold (AuroProbe EM GAM G5: RPN 424; Amersham Biosciences) were used at a dilution of 1:25. Gold particles were enhanced using the IntenSE Silver enhancement kit (RPN 492; Amersham Biosciences) following manufacturer's instructions. Sections were counterstained with uranyl acetate and lead citrate, and examined in a Tecnai 12 electron microscope (FEI). Experiments where primary antibodies were omitted did not produce any specific labelling. The number of particles was quantified in selected regions (see Supplementary Fig. 13a) and the density of gold particles per μm^2^ was calculated. Synapse was defined as a region containing synaptic vesicles clustered at dense projections at the presynaptic membrane, the active zone.

### Data availability

The authors declare that all data supporting the findings of this study are available within the article and its Supplementary Information files or are available from the corresponding authors and the first authors upon request.

## Additional information

**How to cite this article**: Wen, P. J. *et al*. Actin dynamics provides membrane tension to merge fusing vesicles into the plasma membrane. *Nat. Commun.* 7:12604 doi: 10.1038/ncomms12604 (2016).

## Supplementary Material

Supplementary Information Supplementary Figures 1 - 15 and Supplementary References

## References

[b1] Alabi A. A. & Tsien R. W. Perspectives on kiss-and-run: role in exocytosis, endocytosis, and neurotransmission. Annu. Rev. Physiol. 75, 393–422 (2013). 23245563 10.1146/annurev-physiol-020911-153305

[b2] Wu L. G., Hamid E., Shin W. & Chiang H. C. Exocytosis and endocytosis: modes, functions, and coupling mechanisms. Annu. Rev. Physiol. 76, 301–331 (2014). 24274740 10.1146/annurev-physiol-021113-170305 PMC4880020

[b3] Saheki Y. & De Camilli P. Synaptic vesicle endocytosis. Cold Spring Harb. Perspect. Biol. 4, a005645 (2012). 22763746 10.1101/cshperspect.a005645 PMC3428771

[b4] Jahn R. & Fasshauer D. Molecular machines governing exocytosis of synaptic vesicles. Nature 490, 201–207 (2012). 23060190 10.1038/nature11320 PMC4461657

[b5] Sokac A. M., Co C., Taunton J. & Bement W. Cdc42-dependent actin polymerization during compensatory endocytosis in Xenopus eggs. Nat. Cell Biol. 5, 727–732 (2003). 12872130 10.1038/ncb1025

[b6] Nightingale T. D. . Actomyosin II contractility expels von Willebrand factor from Weibel-Palade bodies during exocytosis. J. Cell Biol. 194, 613–629 (2011). 21844207 10.1083/jcb.201011119 PMC3160584

[b7] Masedunskas A. . Role for the actomyosin complex in regulated exocytosis revealed by intravital microscopy. Proc. Natl Acad. Sci. USA 108, 13552–13557 (2011). 21808006 10.1073/pnas.1016778108 PMC3158220

[b8] Nemoto T., Kojima T., Oshima A., Bito H. & Kasai H. Stabilization of exocytosis by dynamic F-actin coating of zymogen granules in pancreatic acini. J. Biol. Chem. 279, 37544–37550 (2004). 15184362 10.1074/jbc.M403976200

[b9] Miklavc P., Wittekindt O. H., Felder E. & Dietl P. Ca2+-dependent actin coating of lamellar bodies after exocytotic fusion: a prerequisite for content release or kiss-and-run. Ann. N. Y. Acad. Sci. 1152, 43–52 (2009). 19161375 10.1111/j.1749-6632.2008.03989.x

[b10] Nightingale T. D., Cutler D. F. & Cramer L. P. Actin coats and rings promote regulated exocytosis. Trends Cell Biol. 22, 329–337 (2012). 22543050 10.1016/j.tcb.2012.03.003

[b11] Chiang H. C. . Post-fusion structural changes and their roles in exocytosis and endocytosis of dense-core vesicles. Nat. Commun. 5, 3356 (2014). 24561832 10.1038/ncomms4356 PMC4267856

[b12] Morton W. M., Ayscough K. R. & McLaughlin P. J. Latrunculin alters the actin-monomer subunit interface to prevent polymerization. Nat. Cell Biol. 2, 376–378 (2000). 10854330 10.1038/35014075

[b13] Brown S. S. & Spudich J. A. Mechanism of action of cytochalasin: evidence that it binds to actin filament ends. J. Cell Biol. 88, 487–491 (1981). 6894300 10.1083/jcb.88.3.487 PMC2112756

[b14] Casella J. F., Flanagan M. D. & Lin S. Cytochalasin D inhibits actin polymerization and induces depolymerization of actin filaments formed during platelet shape change. Nature 293, 302–305 (1981). 7196996 10.1038/293302a0

[b15] Peterson J. R. & Golemis E. A. Autoinhibited proteins as promising drug targets. J. Cell Biochem. 93, 68–73 (2004). 15352163 10.1002/jcb.20184

[b16] Rizvi S. A. . Identification and characterization of a small molecule inhibitor of formin-mediated actin assembly. Chem. Biol. 16, 1158–1168 (2009). 19942139 10.1016/j.chembiol.2009.10.006 PMC2784894

[b17] Korn E. D., Carlier M. F. & Pantaloni D. Actin polymerization and ATP hydrolysis. Science 238, 638–644 (1987). 3672117 10.1126/science.3672117

[b18] Cheever T. R. & Ervasti J. M. Actin isoforms in neuronal development and function. Int. Rev. Cell Mol. Biol. 301, 157–213 (2013). 23317819 10.1016/B978-0-12-407704-1.00004-X

[b19] Ebert S. N. . Targeted insertion of the Cre-recombinase gene at the phenylethanolamine n-methyltransferase locus: a new model for studying the developmental distribution of adrenergic cells. Dev. Dyn. 231, 849–858 (2004). 15517585 10.1002/dvdy.20188

[b20] Perrin B. J., Sonnemann K. J. & Ervasti J. M. beta-actin and gamma-actin are each dispensable for auditory hair cell development but required for Stereocilia maintenance. PLoS Genet. 6, e1001158 (2010). 20976199 10.1371/journal.pgen.1001158 PMC2954897

[b21] Lomasney J. W. . Phosphatidylinositol 4,5-bisphosphate binding to the pleckstrin homology domain of phospholipase C-delta1 enhances enzyme activity. J Biol. Chem. 271, 25316–25326 (1996). 8810295 10.1074/jbc.271.41.25316

[b22] Gasman S. . Regulated exocytosis in neuroendocrine cells: a role for subplasmalemmal Cdc42/N-WASP-induced actin filaments. Mol. Biol. Cell 15, 520–531 (2004). 14617808 10.1091/mbc.E03-06-0402 PMC329227

[b23] Wen P. J. . Phosphatidylinositol(4,5)bisphosphate coordinates actin-mediated mobilization and translocation of secretory vesicles to the plasma membrane of chromaffin cells. Nat. Commun. 2, 491 (2011). 21971506 10.1038/ncomms1500

[b24] Gabel M. . Annexin A2-dependent actin bundling promotes secretory granule docking to the plasma membrane and exocytosis. J. Cell Biol. 210, 785–800 (2015). 26323692 10.1083/jcb.201412030 PMC4555831

[b25] Boulant S., Kural C., Zeeh J. C., Ubelmann F. & Kirchhausen T. Actin dynamics counteract membrane tension during clathrin-mediated endocytosis. Nat. Cell Biol. 13, 1124–1131 (2011). 21841790 10.1038/ncb2307 PMC3167020

[b26] Diz-Munoz A. . Control of directed cell migration in vivo by membrane-to-cortex attachment. PLoS Biol. 8, e1000544 (2010). 21151339 10.1371/journal.pbio.1000544 PMC2994655

[b27] Lee L. M. & Liu A. P. A microfluidic pipette array for mechanophenotyping of cancer cells and mechanical gating of mechanosensitive channels. Lab Chip 15, 264–273 (2015). 25361042 10.1039/c4lc01218f PMC4256121

[b28] Tsujita K., Takenawa T. & Itoh T. Feedback regulation between plasma membrane tension and membrane-bending proteins organizes cell polarity during leading edge formation. Nat. Cell Biol. (2015). 10.1038/ncb3162 25938814

[b29] Tan X., Heureaux J. & Liu A. P. Cell spreading area regulates clathrin-coated pit dynamics on micropatterned substrate. Integr. Biol. 7, 1033–1043 (2015). 10.1039/c5ib00111k PMC4558397 26205141

[b30] Shupliakov O. . Impaired recycling of synaptic vesicles after acute perturbation of the presynaptic actin cytoskeleton. Proc. Natl Acad. Sci. USA 99, 14476–14481 (2002). 12381791 10.1073/pnas.212381799 PMC137908

[b31] Bourne J., Morgan J. R. & Pieribone V. A. Actin polymerization regulates clathrin coat maturation during early stages of synaptic vesicle recycling at lamprey synapses. J. Comp. Neurol. 497, 600–609 (2006). 16739194 10.1002/cne.21006

[b32] Hajkova L., Nyman T., Lindberg U. & Karlsson R. Effects of cross-linked profilin:beta/gamma-actin on the dynamics of the microfilament system in cultured cells. Exp. Cell Res. 256, 112–121 (2000). 10739658 10.1006/excr.1999.4786

[b33] Nyman T., Page R., Schutt C. E., Karlsson R. & Lindberg U. A cross-linked profilin-actin heterodimer interferes with elongation at the fast-growing end of F-actin. J. Biol. Chem. 277, 15828–15833 (2002). 11844798 10.1074/jbc.M112195200

[b34] Grenklo S. . A crucial role for profilin-actin in the intracellular motility of Listeria monocytogenes. EMBO Rep. 4, 523–529 (2003). 12776739 10.1038/sj.embor.embor823 PMC1319178

[b35] Bloom O. . Colocalization of synapsin and actin during synaptic vesicle recycling. J. Cell Biol. 161, 737–747 (2003). 12756235 10.1083/jcb.200212140 PMC2199372

[b36] Evergren E. . Intersectin is a negative regulator of dynamin recruitment to the synaptic endocytic zone in the central synapse. J. Neurosci. 27, 379–390 (2007). 17215399 10.1523/JNEUROSCI.4683-06.2007 PMC6672076

[b37] Kasai H., Takahashi N. & Tokumaru H. Distinct initial SNARE configurations underlying the diversity of exocytosis. Physiol. Rev. 92, 1915–1964 (2012). 23073634 10.1152/physrev.00007.2012

[b38] Lindau M. & Alvarez de Toledo G. The fusion pore. Biochim. Biophys. Acta 164, 167–173 (2003). 12914957 10.1016/s0167-4889(03)00085-5

[b39] Sudhof T. C. The synaptic vesicle cycle. Annu. Rev. Neurosci. 27, 509–547 (2004). 15217342 10.1146/annurev.neuro.26.041002.131412

[b40] Jackson M. B. & Chapman E. R. The fusion pores of Ca(2+)-triggered exocytosis. Nat. Struct. Mol. Biol. 15, 684–689 (2008). 18596819 10.1038/nsmb.1449 PMC2914174

[b41] Heuser J. E. Review of electron microscopic evidence favouring vesicle exocytosis as the structural basis for quantal release during synaptic transmission. Quart. J. Exp. Physiol. 74, 1051–1069 (1989). 10.1113/expphysiol.1989.sp003333 2560556

[b42] Heuser J. E. & Reese T. S. Structural changes after transmitter release at the frog neuromuscular junction. J. Cell Biol. 88, 564–580 (1981). 6260814 10.1083/jcb.88.3.564 PMC2112753

[b43] Watanabe S. . Ultrafast endocytosis at *Caenorhabditis elegans* neuromuscular junctions. Elife 2, e00723 (2013). 24015355 10.7554/eLife.00723 PMC3762212

[b44] Malacombe M., Bader M. F. & Gasman S. Exocytosis in neuroendocrine cells: new tasks for actin. Biochim. Biophys. Acta 1763, 1175–1183 2006. 17034880 10.1016/j.bbamcr.2006.09.004

[b45] Porat-Shliom N., Milberg O., Masedunskas A. & Weigert R. Multiple roles for the actin cytoskeleton during regulated exocytosis. Cell Mol. Life Sci. 70, 2099–2121 (2013). 22986507 10.1007/s00018-012-1156-5 PMC4052552

[b46] Berberian K., Torres A. J., Fang Q., Kisler K. & Lindau M. F-actin and myosin II accelerate catecholamine release from chromaffin granules. J. Neurosci. 29, 863–870 (2009). 19158310 10.1523/JNEUROSCI.2818-08.2009 PMC2768403

[b47] Neco P. . Myosin II contributes to fusion pore expansion during exocytosis. J. Biol. Chem. 283, 10949–10957 (2008). 18283106 10.1074/jbc.M709058200

[b48] Olivares M. J. . Src kinases regulate *de novo* actin polymerization during exocytosis in neuroendocrine chromaffin cells. PLoS ONE 9, e99001 (2014). 24901433 10.1371/journal.pone.0099001 PMC4047038

[b49] Fang Q. . The role of the C terminus of the SNARE protein SNAP-25 in fusion pore opening and a model for fusion pore mechanics. Proc. Natl Acad. Sci. USA 105, 15388–15392 (2008). 18829435 10.1073/pnas.0805377105 PMC2563113

[b50] Doreian B. W., Fulop T. G. & Smith C. B. Myosin II activation and actin reorganization regulate the mode of quantal exocytosis in mouse adrenal chromaffin cells. J. Neurosci. 28, 4470–4478 (2008). 18434525 10.1523/JNEUROSCI.0008-08.2008 PMC2745116

[b51] Klyachko V. A. & Jackson M. B. Capacitance steps and fusion pores of small and large-dense-core vesicles in nerve terminals. Nature 418, 89–92 (2002). 12097912 10.1038/nature00852

[b52] He L., Wu X. S., Mohan R. & Wu L. G. Two modes of fusion pore opening revealed by cell-attached recordings at a synapse. Nature 444, 102–105 (2006). 17065984 10.1038/nature05250

[b53] Rangaraju V., Calloway N. & Ryan T. A. Activity-driven local ATP synthesis is required for synaptic function. Cell 156, 825–835 (2014). 24529383 10.1016/j.cell.2013.12.042 PMC3955179

[b54] Hroudova J., Singh N. & Fisar Z. Mitochondrial dysfunctions in neurodegenerative diseases: relevance to Alzheimer's disease. Biomed. Res. Int. 2014, 175062 (2014). 24900954 10.1155/2014/175062 PMC4036420

[b55] Lian G. & Sheen V. L. Cytoskeletal proteins in cortical development and disease: actin associated proteins in periventricular heterotopia. Front. Cell Neurosci. 9, 99 (2015). 25883548 10.3389/fncel.2015.00099 PMC4381626

[b56] Lindau M. & Neher E. Patch-clamp techniques for time-resolved capacitance measurements in single cells. Pflugers Arch. 411, 137–146 (1988). 3357753 10.1007/BF00582306

[b57] Heidelberger R. ATP is required at an early step in compensatory endocytosis in synaptic terminals. J. Neurosci. 21, 6467–6474 (2001). 11517235 10.1523/JNEUROSCI.21-17-06467.2001 PMC6763084

[b58] Augustine G. J. & Neher E. Calcium requirements for secretion in bovine chromaffin cells. J. Physiol. 450, 247–271 (1992). 1432709 10.1113/jphysiol.1992.sp019126 PMC1176121

[b59] Elhamdani A., Azizi F. & Artalejo C. R. Double patch clamp reveals that transient fusion (kiss-and-run) is a major mechanism of secretion in calf adrenal chromaffin cells: high calcium shifts the mechanism from kiss-and-run to complete fusion. J. Neurosci. 26, 3030–3036 (2006). 16540581 10.1523/JNEUROSCI.5275-05.2006 PMC6673983

[b60] Artalejo C. R., Adams M. E. & Fox A. P. Three types of Ca^2+^ channel trigger secretion with different efficacies in chromaffin cells. Nature 367, 72–76 (1994). 8107778 10.1038/367072a0

[b61] Artalejo C. R., Henley J. R., McNiven M. A. & Palfrey H. C. Rapic endocytosis coupled to exocytosis in adrenal chromaffin cells involves Ca^2+^, GTP, and dynamin but not clathrin. Proc. Natl Acad. Sci. USA 92, 8328–8332 (1995). 7667289 10.1073/pnas.92.18.8328 PMC41150

[b62] Artalejo C. R., Elhamdani A. & Palfrey H. C. Calmodulin is the divalent cation receptor for rapid endocytosis, but not exocytosis, in adrenal chromaffin cells. Neuron 16, 195–205 (1996). 8562084 10.1016/s0896-6273(00)80036-7

[b63] Shawlot W., Deng J. M., Fohn L. E. & Behringer R. R. Restricted beta-galactosidase expression of a hygromycin-lacZ gene targeted to the beta-actin locus and embryonic lethality of beta-actin mutant mice. Transgenic Res. 7, 95–103 (1998). 9608737 10.1023/a:1008816308171

[b64] Shmerling D. . Strong and ubiquitous expression of transgenes targeted into the beta-actin locus by Cre/lox cassette replacement. Genesis 42, 229–235 (2005). 16028230 10.1002/gene.20135

[b65] Wu X. S. . Calcineurin is universally involved in vesicle endocytosis at neuronal and nonneuronal secretory cells. Cell Rep. 7, 982–988 (2014). 24835995 10.1016/j.celrep.2014.04.020 PMC4070379

[b66] Smith C. & Neher E. Multiple forms of endocytosis in bovine adrenal chromaffin cells. J. Cell Biol. 139, 885–894 (1997). 9362507 10.1083/jcb.139.4.885 PMC2139962

[b67] Gad H. . Fission and uncoating of synaptic clathrin-coated vesicles are perturbed by disruption of interactions with the SH3 domain of endophilin. Neuron 27, 301–312 (2000). 10985350 10.1016/s0896-6273(00)00038-6

