# Supplementary material for: Actin dynamics provides membrane tension to merge fusing vesicles into the plasma membrane
Source: Nat Commun. 2016 Aug 31;7:12604. doi: 10.1038/ncomms12604 (PMC5013665; doi:10.1038/ncomms12604)
Supplement: Supplementary Information — Supplementary Figures 1 - 15 and Supplementary References [file ncomms12604-s1.pdf]

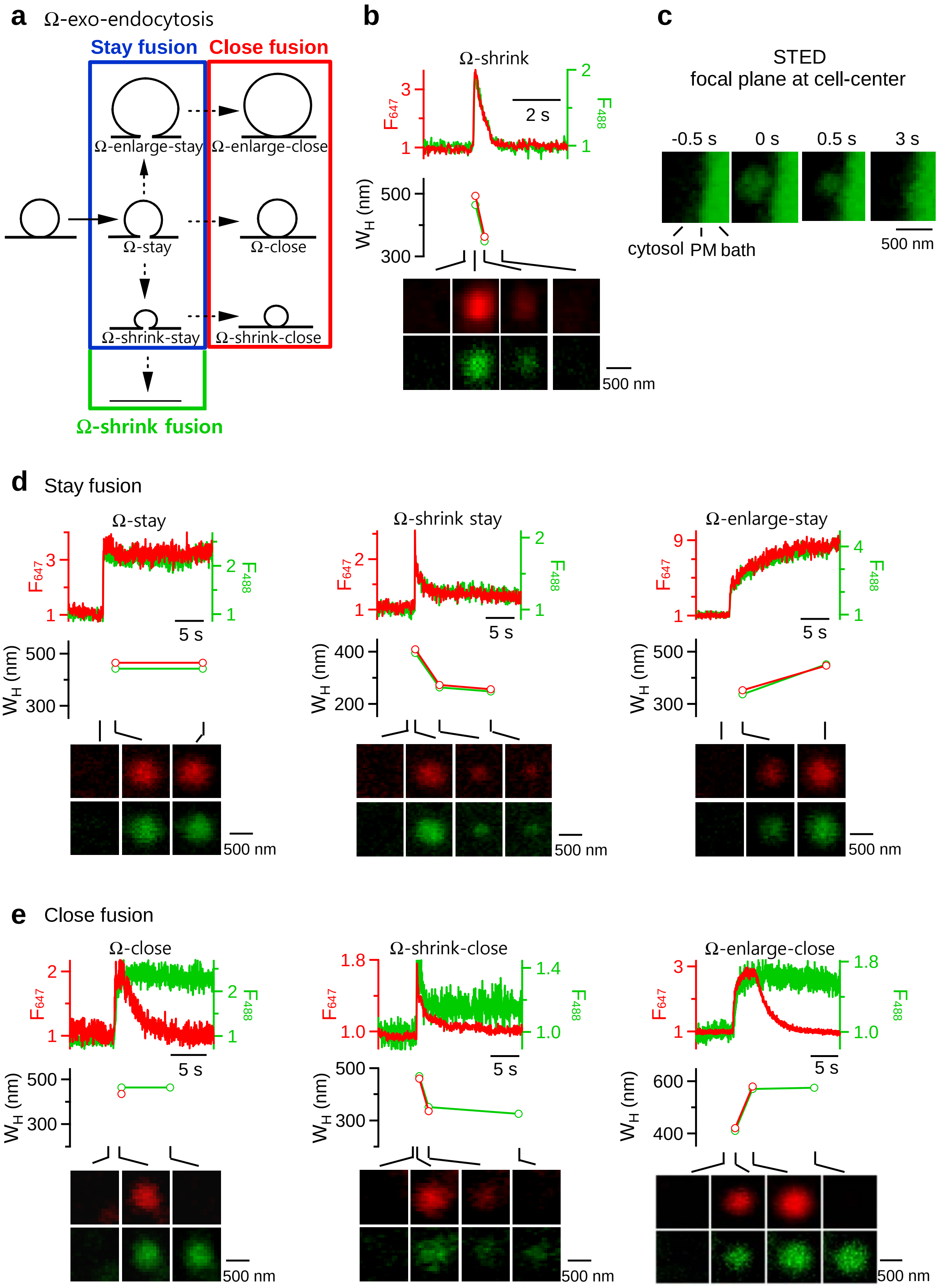
a
Ω-exo-endocytosis
Stay fusion
Close fusion
Ω-enlarge-stay
Ω-enlarge-close
Ω-stay
Ω-close
Ω-shrink-stay
Ω-shrink-close
Ω-shrink fusion
b
Ω-shrink
2 s
500 nm
c
STED
focal plane at cell-center
-0.5 s
0 s
0.5 s
3 s
cytosol
PM
bath
500 nm
d
Stay fusion
Ω-stay
Ω-shrink stay
Ω-enlarge-stay
5 s
500 nm
e
Close fusion
Ω-close
Ω-shrink-close
Ω-enlarge-close
5 s
500 nm

1

Supplementary Figure 1. Seven fusion modes can be summarized into three categories: Ω-shrink, stay and close fusion in bovine chromaffin cells

(a) Schematic drawings of a model called Ω-exo-endocytosis with seven fusion modes, the name of which is written under each configuration. These seven fusion modes can be regrouped into three categories, Ω-shrink, stay, and close fusion as described below. The dotted arrows indicated the events may or may not take place.

1. Ω-shrink fusion (green box) refers to a fusion mode in which the fusion-generated Ω-profile shrinks until undetectable.

2. Stay fusion (blue box) refers to three individual fusion modes, in which the fusion-generated Ω-profiles are maintained with an open pore while the size of the Ω-profile may change. These three individual fusion modes are Ω-stay, Ω-enlarge-stay, and Ω-shrink-stay fusion, as defined below.

Ω-stay: the Ω-profile remains unchanged in its size with an opened pore after fusion.

Ω-shrink-stay: the Ω-profile shrinks to some extent with an opened pore.

Ω-enlarge-stay: the Ω-profile enlarges with an opened pore.

3. Close fusion (red box) refers to stay fusion that is followed by pore closure. It includes three individual fusion modes, Ω-close, Ω-enlarge-close, and Ω-shrink-close, as defined below.

Ω-close: the fusion-generated Ω-profile remains unchanged in its size but closes its pore sometime after fusion.

Ω-shrink-close: the fusion-generated Ω-profile shrinks its size to some extent and closes its pore sometime after fusion.

Ω-enlarge-close: the fusion-generated Ω-profile enlarges its size and closes its pore sometime after fusion.

(b) Ω-shrink: A647 fluorescence intensity (F_{647}, red), A488 fluorescence intensity (F_{488}, green), W_H, and sampled images (average of 4 frames) at times indicated (lines), are plotted versus time for a spot undergoing Ω-shrink fusion induced by $Train_{2Hz}$. The images were collected every 20-40 ms at the confocal cell-bottom setting with A647 and A488 in the bath excited strongly and weakly, respectively. F_{647} or F_{488} was normalized to its mean background value before spots appeared. This plot is identical to Fig. 1c. The settings of this plot also apply to panels d-e.

(c) Sampled Ω-shrink fusion observed at a focal plane of the STED microscope above the cell-bottom (>2 μm) where the plasma membrane was approximately in parallel with the microscopic z axis (side view). The time is relative to the time the spot appeared. The bath containing fluorescent A488, the cytosol (dark), and the plasma membrane (PM) are labelled in the first panel.

(d) Stay fusion: F_{647}, F_{488}, W_H, and sampled images (average of 5-20 frames) at times indicated (lines) are plotted versus time for three spots undergoing Ω-stay (left), Ω-shrink-stay (middle), and Ω-enlarge-stay (right) fusion, as induced by $Train_{2Hz}$.

(e) Close fusion: F_{647}, F_{488}, W_H, and sampled images (average of 10-16 frames) at times indicated (lines) are plotted versus time for three spots undergoing Ω-close (left), Ω-shrink-close (middle), and Ω-enlarge-close (right) fusion.

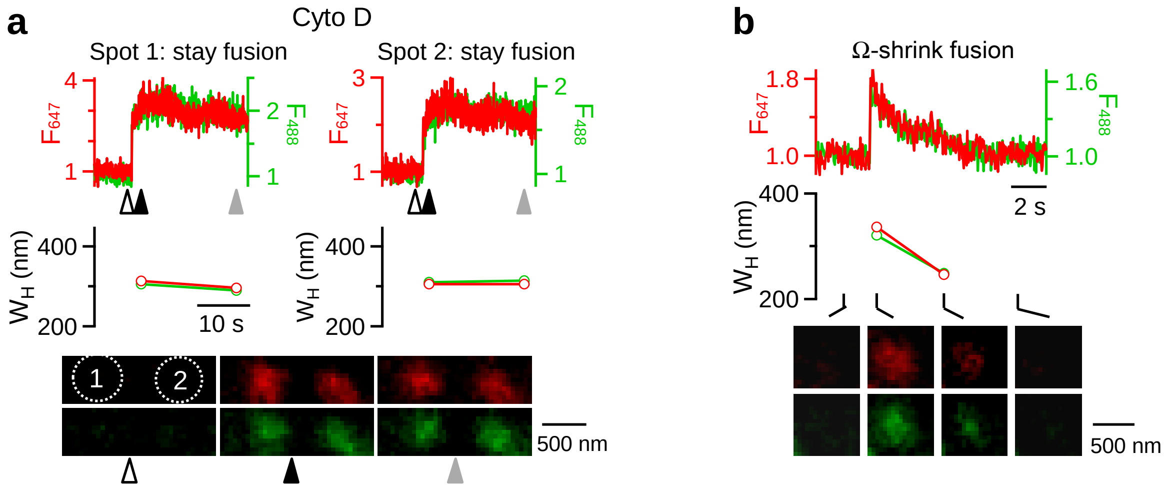

Supplementary Figure 2. Cytochalasin D inhibits Ω-shrink fusion

(a) An example showing more spots (two neighbouring spots, 1 & 2) undergoing stay fusion during $Train_{2Hz}$ in the presence of cytochalasin D (Cyto D, 4 μM, in bath solution). Sampled images were taken at times indicated by triangles.

(b) A spot undergoing Ω-shrink fusion in the presence of Cyto D – shrinking rate is slower compared to control in Fig. 1d, 1g in the main text.

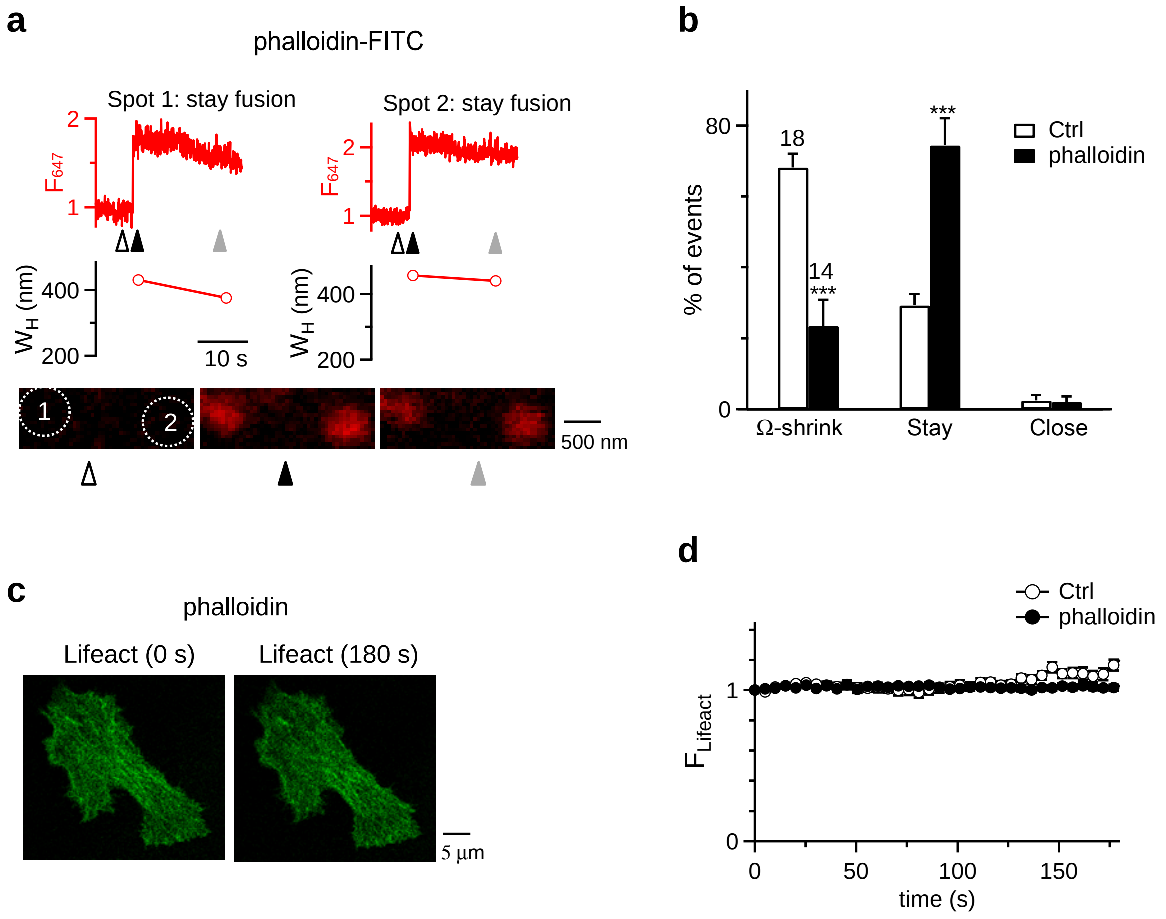

Supplementary Figure 3. Phalloidin inhibits Ω-shrink fusion

(a) An example showing more spots (two neighbouring spots 1 and 2) undergoing stay fusion during $Train_{2Hz}$ in the presence of phalloidin-FITC (1.3 µM in the pipette, 2-3 min after whole-cell dialysis). F_{647} (red) and W_H and sampled images taken at times indicated by triangles are shown.

(b) Bar graph showing percentages (mean + s.e.m.) of Ω-shrink, stay and close fusion induced by $Train_{2Hz}$ in the presence of 1.3 µM phalloidin-FITC (n=14 cells; total spot number: 111). Control is plotted for comparison (white, same as Fig. 1h, n=18 cells, total spot number: 192 spots). The cell number is also shown at the top of the corresponding bar (applies to other similar plots). ***: $P < 0.001$, unpaired two-tailed Student's t-test.

(c) Sampled Lifeact images at the cell-bottom before (0 s, left) and 180 s after (right) whole-cell dialysis of phalloidin (1.3 µM).

(d) Lifeact-TagGFP2 fluorescence ($F_{Lifeact}$, mean ± s.e.m.) at the cell-bottom before (time 0) and during whole-cell dialysis of phalloidin (1.3 µM, 7 cells). $F_{Lifeact}$ was normalized to the value at time 0.

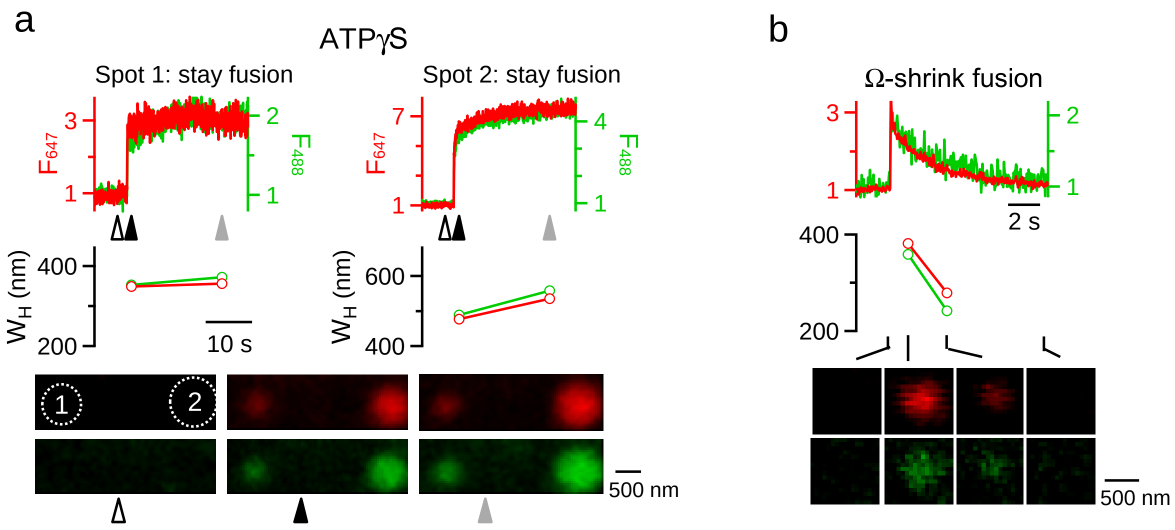

Supplementary Figure 4. ATPγS inhibits Ω-shrink fusion

(a) An example showing more spots (two neighbouring spots, 1 & 2) undergoing stay fusion during Train$_{2Hz}$ in the presence of ATPγS (2 mM, replacing 2 mM ATP in the pipette). Sampled images were taken as indicated by triangles.

(b) A spot undergoing Ω-shrink fusion in the presence of ATPγS – shrinking rate is slower compared to control in Fig. 1d, 1g in the main text.

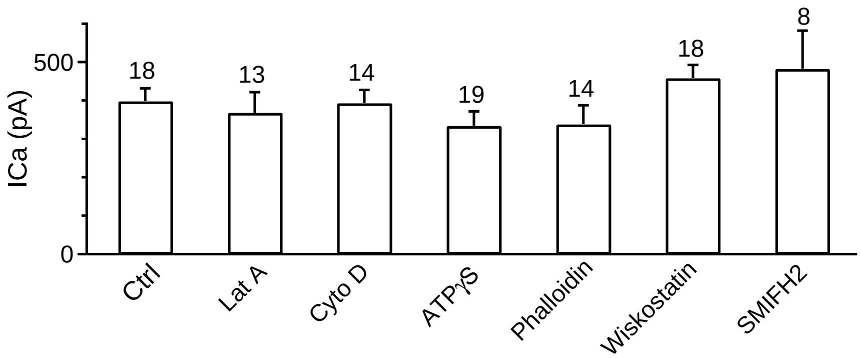

Supplementary Figure 5. Lat A, Cyto D, ATPγS, phalloidin, wiskostatin or SMIFH2 treatment does not affect ICa

The amplitude (mean + s.e.m) of the ICa induced by $Train_{2Hz}$ in control (n = 18 cells), in the presence of Lat A (3 µM, n = 13 cells, $P = 0.6151$), cytochalasin D (Cyto D, 4 µM, n = 9 cells, $P = 0.9171$), ATPγS (2 mM, n = 19 cells, $P = 0.2039$), phalloidin-FITC (1.3 µM, n = 14 cells, $P = 0.292$), wiskostatin (10 µM, n = 18 cells, $P = 0.2114$) or SMIFH2 (25 µM, n = 8 cells, $P = 0.1323$). ICa amplitude was measured from the first 50 ms pulse during $Train_{2Hz}$. Lat A, Cyto D, wiskostatin and SMIFH2 were applied to the bath for 20 min before ICa was measured. Phalloidin-FITC and ATPγS was dialyzed into the cell for ~2 min via a whole-cell pipette. All measurements were made within ~2-3 min after whole-cell break-in. They were not significantly different ($P > 0.05$; unpaired two-tailed Student's t-test).

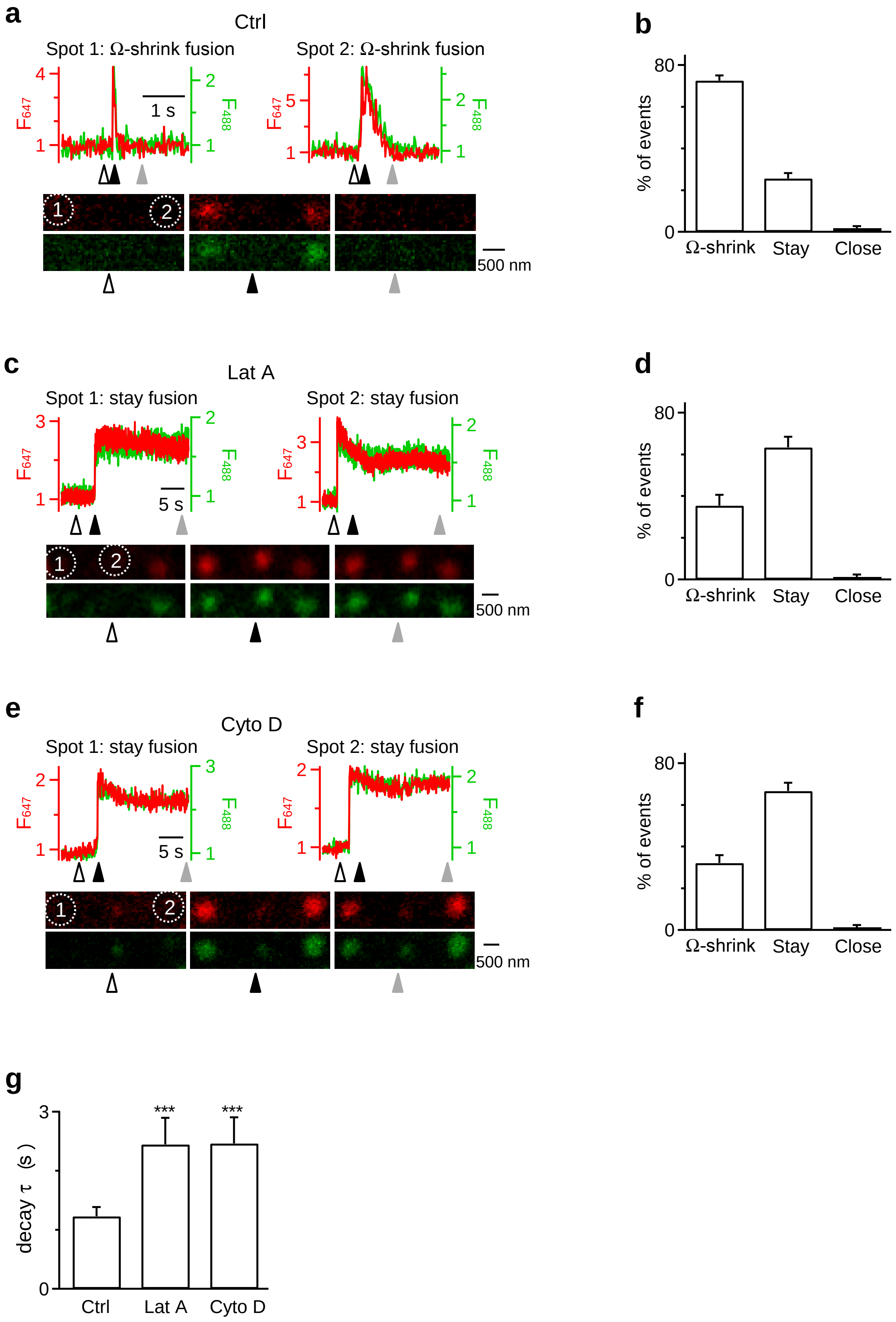
a
Ctrl
Spot 1: Ω-shrink fusion
Spot 2: Ω-shrink fusion
F647
F488
1 s
500 nm
b
% of events
Ω-shrink
Stay
Close
c
Lat A
Spot 1: stay fusion
Spot 2: stay fusion
5 s
d
e
Cyto D
f
g
decay τ (s)
Ctrl
Lat A
Cyto D
***

114

Supplementary Figure 6. Lat A and Cyto D inhibit Ω-shrink fusion, but promote stay fusion during intracellular calcium dialysis

(a) An example showing that Ω-shrink fusion is the majority of fusion events induced by calcium (1.5 μM) dialysis in control: F_{647}, F_{488}, W_H, and sampled images for two neighbouring spots (1 and 2) undergoing Ω-shrink fusion during calcium dialysis. Data were taken within 2 min after calcium dialysis (applies to all panels in this figure). Triangles indicate the time, at which sampled images were taken.

(b) The percentages (mean + s.e.m.) of Ω-shrink, stay and close fusion induced by calcium dialysis in control (n=23 cells, total spot number: 399).

(c-d) Similar arrangements as in panels a-b, respectively, except that Lat A (3 μM) was added in the bath solution for 20 min (n=17 cells, total spot number: 157). c: two spots underwent stay fusion.

(e-f) Similar arrangements as in panels a-b, respectively, except that Cyto D (4 μM) was added in the bath solution for 20 min (n=18 cells, total spot number: 157). e: two spots underwent stay fusion.

(g) F_{647} decay time constant (τ, mean + s.e.m.) for Ω-shrink fusion in control (n=276 spots), and in the presence of Lat A (n=55 spots) or Cyto D (n=51 spots). ***: $P < 0.001$; unpaired two-tailed Student's t-test.

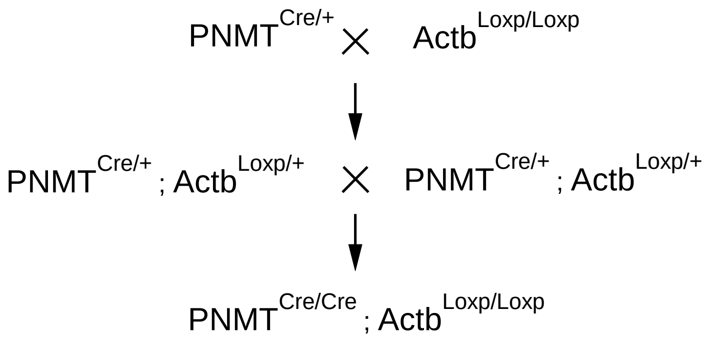

Supplementary Figure 7. Breeding protocol for generating $PNMT^{Cre/Cre}$; $Actb^{LoxP/LoxP}$ mice ($Actb^{-/-}$ mice)

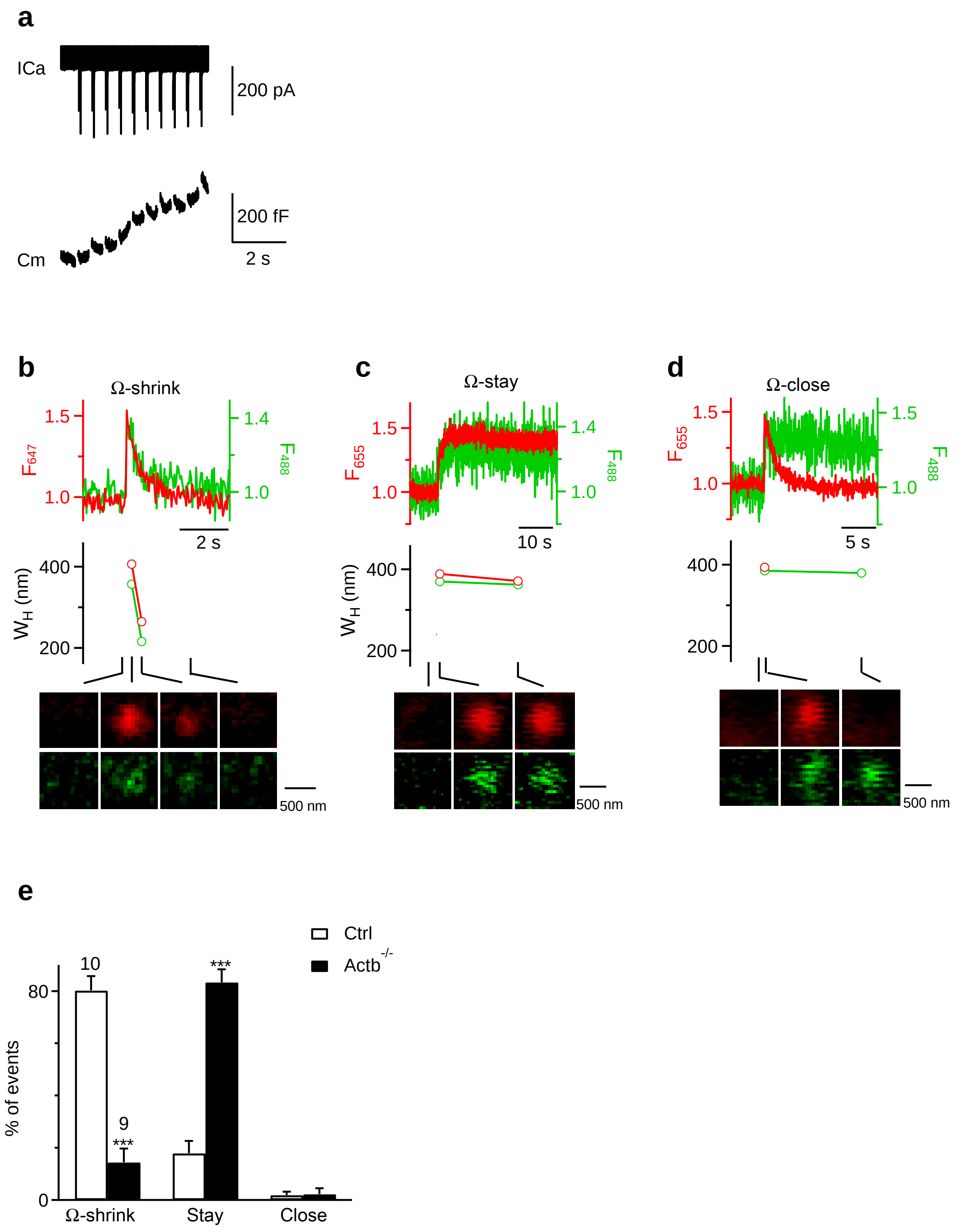
a
ICa
200 pA
200 fF
2 s
Cm
b
Ω-shrink
1.5
1.0
1.4
1.0
2 s
400
200
500 nm
c
Ω-stay
1.5
1.0
1.4
1.0
10 s
400
200
500 nm
d
Ω-close
1.5
1.0
1.5
1.0
5 s
400
200
500 nm
e
Ctrl
Actb-/-
10
9
***
***
80
0
% of events
Ω-shrink
Stay
Close

Supplementary Figure 8. Three fusion categories in control and $Actb^{-/-}$ mouse chromaffin cells induced by $Train_{2Hz}$

(a) Sampled ICa and the Cm change induced by $Train_{2Hz}$ in a wild-type mouse chromaffin cell

(b) **Ω**-shrink: A647 fluorescence intensity (F_{647}, red), A488 fluorescence intensity (F_{488}, green), W_H, and sampled images (average of 42 frames) at times indicated (lines), are plotted versus time for a spot undergoing **Ω**-shrink fusion induced by $Train_{2Hz}$ in a wild-type mouse chromaffin cell. The images were collected every 40 ms at the confocal cell-bottom setting with A647 and A488 in the bath excited strongly and weakly, respectively. F_{647} or F_{488} was normalized to its mean background value before spots appeared.

(c) Stay fusion: F_{647}, F_{488}, W_H, and sampled images (average of 10-16 frames) at times indicated (lines) are plotted versus time for a spot undergoing stay fusion in a wild-type mouse chromaffin cell.

(d) Close fusion: F_{647}, F_{488}, W_H, and sampled images (average of 10-16 frames) at times indicated (lines) are plotted versus time for a spot undergoing close fusion in a wild-type mouse chromaffin cell.

(e) Percentages (mean + s.e.m.) of **Ω**-shrink, stay and close fusion induced by $Train_{2Hz}$ in control (n=10 cells; total spot number: 80) and $Actb^{-/-}$ cells (n=9 cells; total spot number: 56; ***: $P < 0.001$, two-tailed Student's t test).

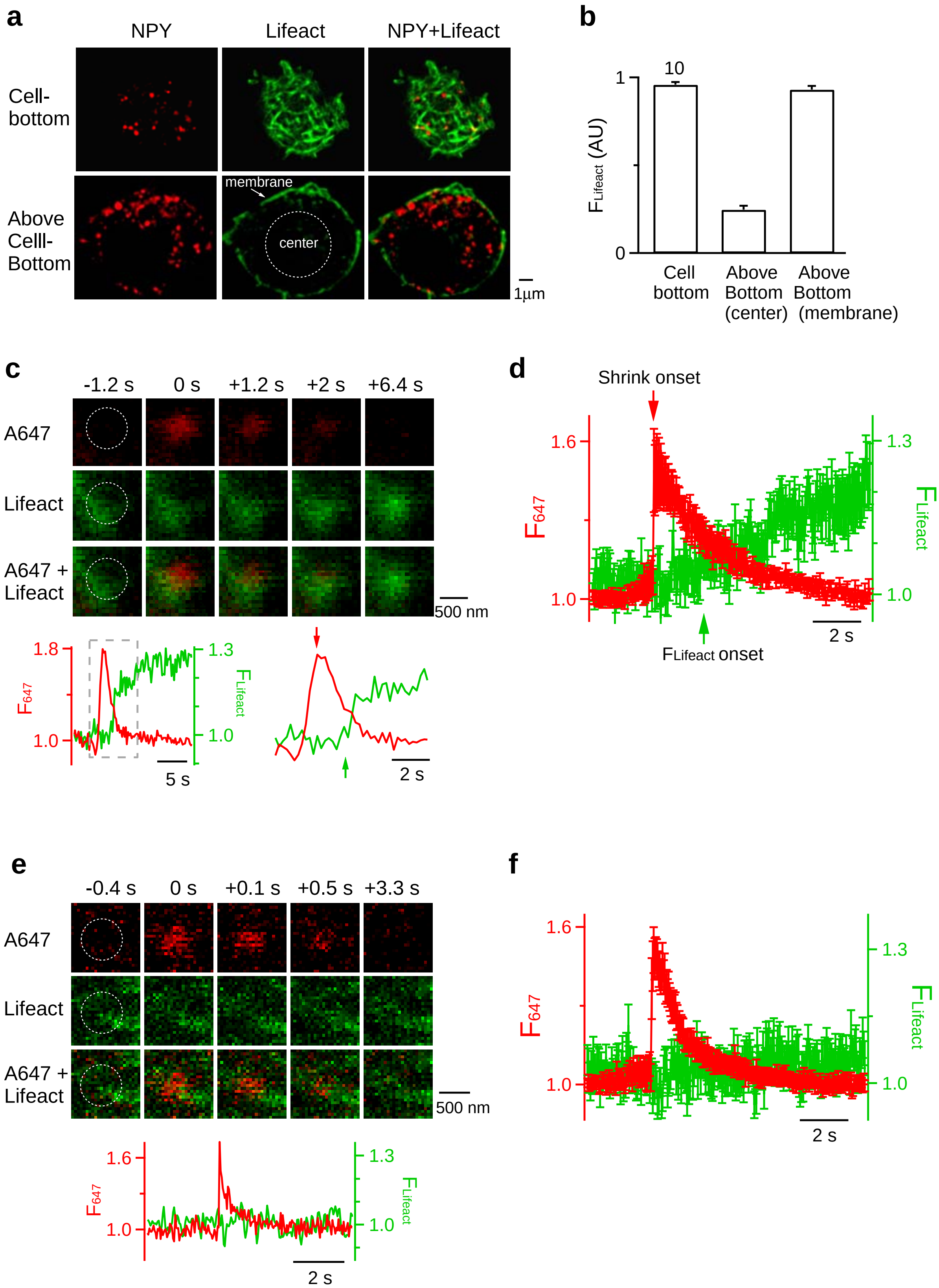
a
NPY
Lifeact
NPY+Lifeact
Cell-
bottom
Above
Celll-
Bottom
membrane
center
1μm
b
10
1
0
FLifeact (AU)
Cell
bottom
Above
Bottom
(center)
Above
Bottom
(membrane)
c
-1.2 s
0 s
+1.2 s
+2 s
+6.4 s
A647
Lifeact
A647 +
Lifeact
500 nm
1.8
1.0
F647
1.3
1.0
FLifeact
5 s
2 s
d
Shrink onset
1.6
1.0
F647
1.3
1.0
FLifeact
2 s
FLifeact onset
e
-0.4 s
0 s
+0.1 s
+0.5 s
+3.3 s
A647
Lifeact
A647 +
Lifeact
500 nm
1.6
1.0
F647
1.3
1.0
FLifeact
2 s
f
1.6
1.0
F647
1.3
1.0
FLifeact
2 s

Supplementary Figure 9. Dense F-actin network at the plasma membrane does not move, or move with a delay, towards the shrinking Ω-profile

(a) Sampled STED images of a cell overexpressing NPY-mCherry (left, red) and Lifeact (middle, green) at the cell-bottom (upper panels) and above cell-bottom (~2.5 μm above, lower panels). Left and middle images are superimposed in the right.

(b) $F_{Lifeact}$ (mean + s.e.m.) at the cell-bottom membrane (10 cells, e.g., panel a, upper image), above-cell-bottom center region at least 1 μm away from plasma membrane (10 cells, e.g., dotted circle in panel a) and above-cell-bottom membrane (10 cells, e.g., arrow in panel a).

(c) Images: simultaneous confocal imaging of A647 (bath, top) and Lifeact (middle, Lifeact was overexpressed) at the cell-bottom shows a gradual increase of Lifeact fluorescence (actin increases) as the A647 spot shrank. Top and middle panels are superimposed in the bottom (applies to panel e). The times labelled are relative to the time at which the A647 spot reached the peak intensity (defined as time 0).

Traces: F_{647} and $F_{Lifeact}$ of the dotted circle indicated in the upper panel are plotted versus time (left). The dotted box in left is expanded in the right to indicate a delay of $F_{Lifeact}$ increase (green arrow) as compared to F_{647} decrease (red arrow).

(d) The averaged F_{647} and $F_{Lifeact}$ from 15 fusion spots undergoing Ω-shrink fusion (mean ± s.e.m). The traces were aligned at the onset of A647 spot appearance. Arrows indicate $F_{Lifeact}$ increase (green arrow) is delayed as compared to F_{647} decrease (red arrow).

(e-f) Similar to panel c-d, respectively, but for Ω-shrink fusion spots with no clear $F_{Lifeact}$ increase. f, n = 22 spots.

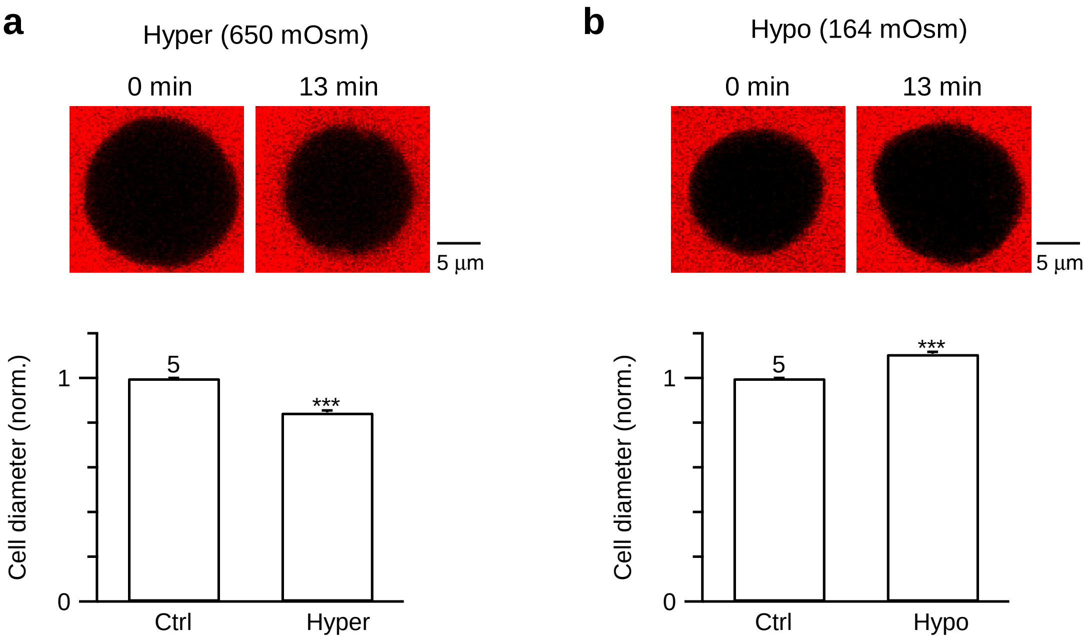

Supplementary Figure 10. Hyper-osmotic and hypo-osmotic solution shrinks and enlarges chromaffin cells, respectively

Cells shrink or enlarge in hyper-osmotic (650 mOsm, Hyper) or hypo-osmotic (164 mOsm) solution, respectively

(a) Upper: A sampled cell imaged at the cell-center (>2 µm above cell-bottom) before (0 min, 305 mOsm) and 13 min after application of the hyper-osmotic solution (Hyper, 650 mOsm). A647 was included in the bath solution for labeling cell outline.

Lower: Cell diameter in control (Ctrl, before hyper-osmotic application, 305 mOsm) and in hyper-osmotic solution (Hyper, 650 mOsm). Data are normalized to the Ctrl and expressed as mean + s.e.m. (n = 5 cells, ***: $P < 0.001$, two-tailed paired Student's t-test).

(b) Similar to panel a, except that the hyper-osmotic solution was replaced with hypo-osmotic solution (Hypo, 164 mOsm). Data are normalized to the Ctrl and expressed as mean + s.e.m. (n = 5 cells, ***: $P < 0.001$, two-tailed paired Student's t-test).

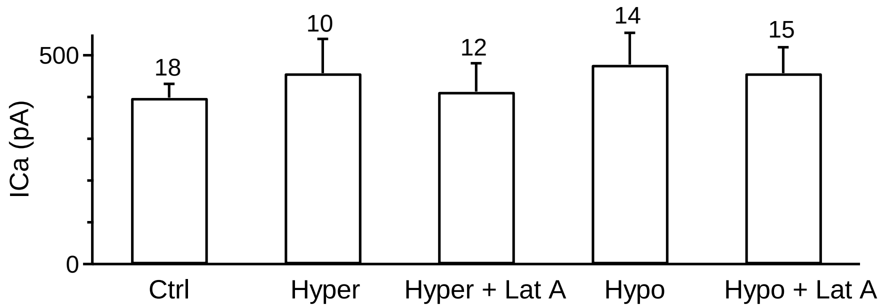

Supplementary Figure 11. Changes in the osmolarity of extracellular solution do not affect ICa

The amplitude (mean + s.e.m) of the ICa induced by $Train_{2Hz}$ in control (Ctrl, n = 18 cells), hyper-osmotic solution (Hyper, 650 Osm, n = 10 cells, $P = 0.4264$), hyper-osmotic solution plus Lat A (Hyper + Lat A, n = 12 cells, $P = 0.8252$), hypo-osmotic solution (Hypo, 164 Osm, n = 14 cells, $P = 0.3028$), hypo-osmotic solution plus Lat A (Hypo + Lat A, n = 15 cells, $P = 0.3763$). ICa amplitude was measured from the first 50 ms pulse during $Train_{2Hz}$. When Lat A (3 μM) was added, cells were pre-treated for at least 10 min before exposed to hyper-osmotic or hypo-osmotic solution in the presence of Lat A. Statistical significances were assessed by unpaired two-tailed Student's t-test.

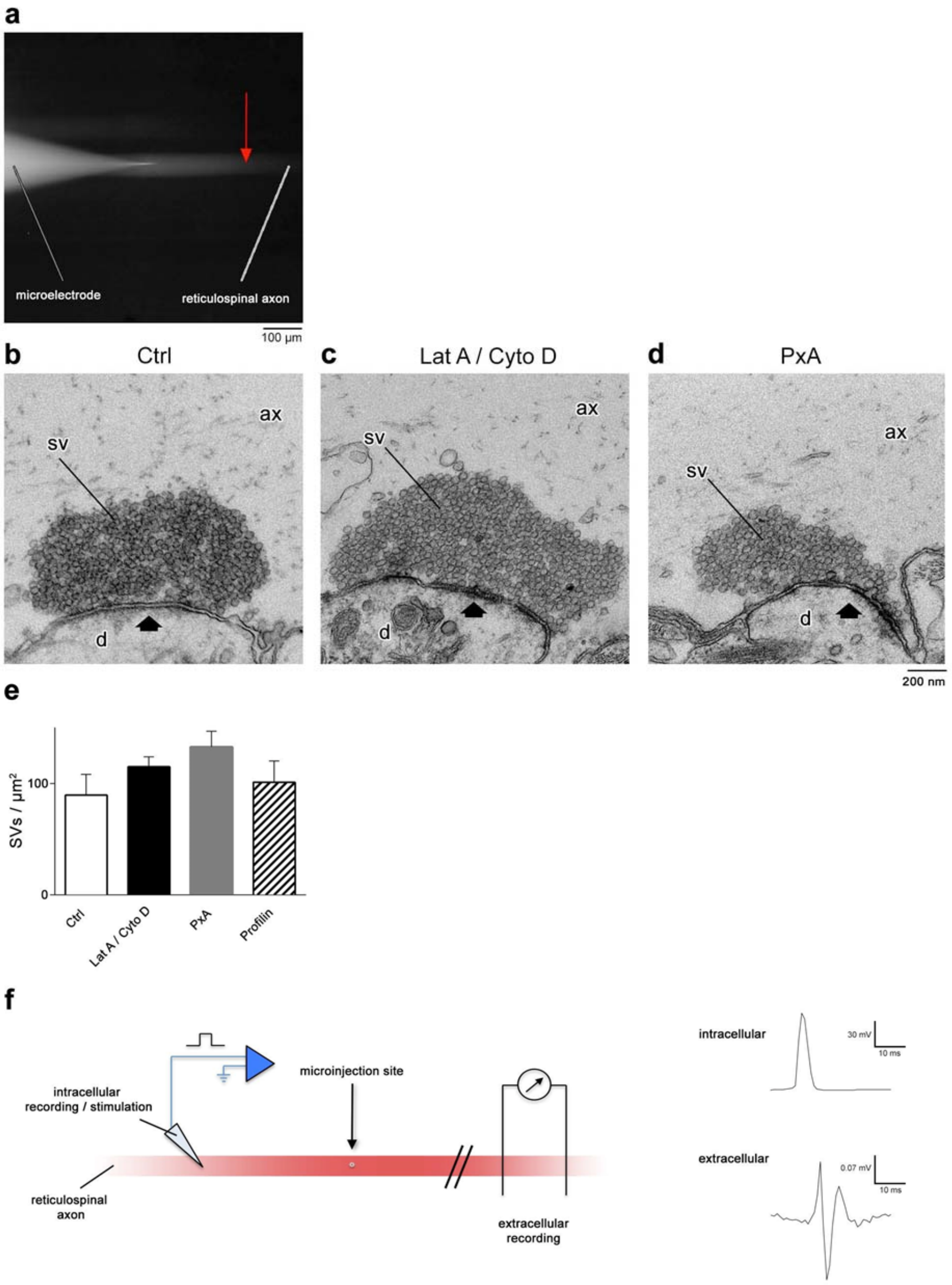
a
microelectrode
reticulospinal axon
100 µm
b
Ctrl
c
Lat A / Cyto D
d
PxA
ax
sv
d
200 nm
e
SVs / µm²
100
0
Ctrl
Lat A / Cyto D
PxA
Profilin
f
intracellular recording / stimulation
microinjection site
reticulospinal axon
extracellular recording
intracellular
30 mV
10 ms
extracellular
0.07 mV
10 ms

Supplementary Figure 12. Microinjections of actin-directed compounds did not affect active zone morphology or action potential propagation

(a) A CCD camera image of a reticulospinal axon in the lamprey spinal cord microinjected with Lat A/Cyto D and Texas Red. The axon was labeled with Texas Red (horizontal cylinder like structure as indicated). Texas Red fluorescence intensity decayed gradually from left, the injection site), to the right along the axon. Red arrow indicates the place where we examined the tissue at the EM level. At this location, Texas Red fluorescence intensity was ~10 times lower than in the tip of micropipette, suggesting that the concentration of Lat A/Cyto D was ~10 times lower than in the micropipette.

(b-d) Electron microscopic images of vesicle clustering at the active zone of a control axon (b), an axon microinjected with Lat A/Cyto D (c), or an axon microinjected with PxA (d). Axons were stimulated at 5 Hz for 30 min. Note normal morphology of the axoplasmic matrix (ax) and an accumulation of synaptic vesicles (sv) at active zones (thick arrows) and clathrin-coated pits around the active zone in (b). d, postsynaptic dendrite. The active zone with Ω–shape profiles from the synapse shown in (d) is depicted in Fig. 6e at higher magnification.

(e) Bar graph showing the number of vesicles at the active zone (AZ) within 50 nm from the presynaptic membrane in control axons (no microinjection) and in axons microinjected with Lat A/Cyto D, PxA or profilin I. Data were plotted as (mean + s.e.m.)/(μm^2 AZ). The axon was fixed after action potential stimulation at 5 Hz for 30 min. Each group of data was from 5 synapses cut in serial section from 3 microinjected axons. The one-way analysis of variance (ANOVA) was used to test statistical difference between the means, alpha level of 0.05, followed by Holm- Šídák's test. Differences between the means were not significant. $P > 0.05$ ($P = 0.7184$ for Ctrl vs. Lat A/Cyto D, $P = 0.3551$ for Ctrl vs. PxA, $P = 0.8206$ for Ctrl vs. profilin).

(f) Left: schematics illustrating an experiment, in which the ability of a reticulospinal axon to propagate actin potentials following microinjection of a mixture of Lat A/Cyto D and Texas Red was tested. Action potential was induced by intracellular stimulation of axons rostral to the microinjection (intracellular recording/stimulation).

Right: Action potential recorded intracellularly (upper) successfully propagated through the site of microinjections and were recorded extracellularly at 5 cm away at the caudal part of the spinal cord preparation (extracellular recording), confirming that microinjection of Lat A/Cyto D did not affect action potential propagation along the axon.

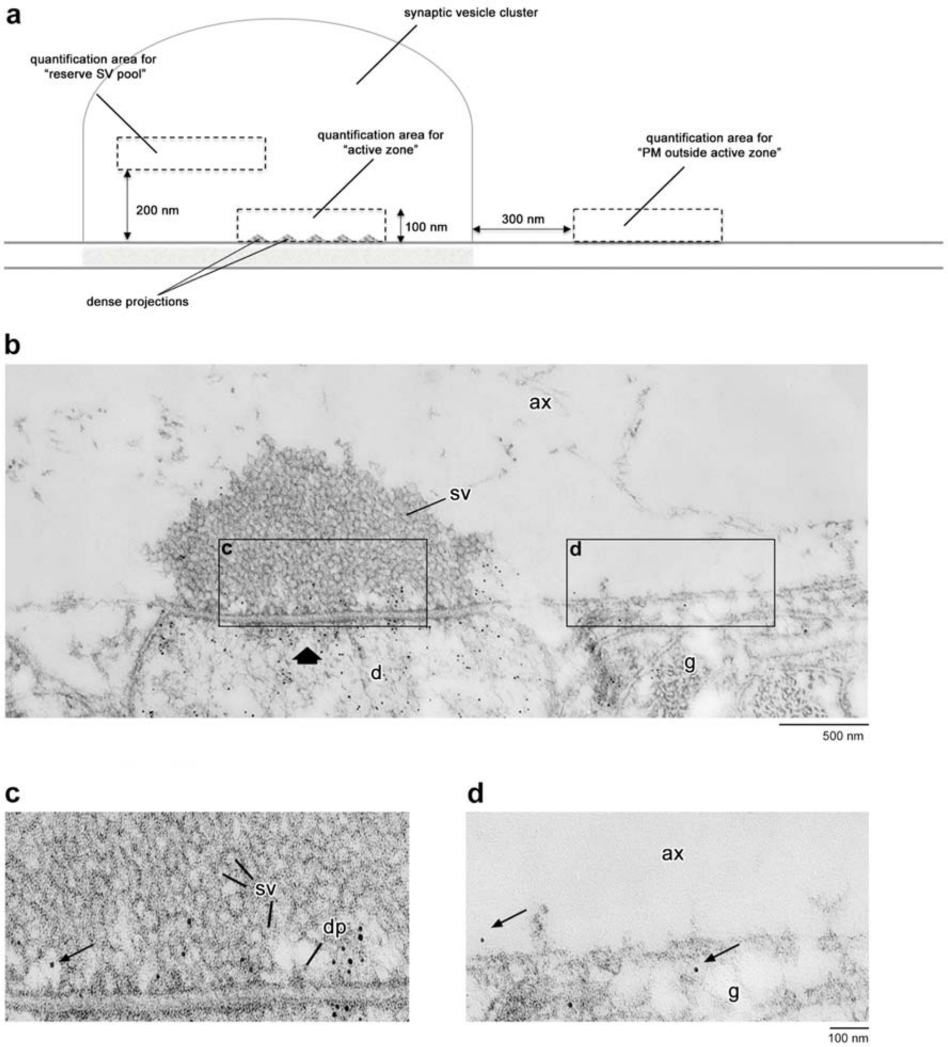

Supplementary Figure 13. Quantification of immunogold labeling of actin at the giant reticulospinal synapse in lamprey

(a) Schematics showing three areas where we quantified actin immunogold particles: active zone, PM outside active zone, and the reserve pool. Results of quantifications are shown in Fig. 6j.

(b) Electron micrograph of a giant reticulospinal synapse labeled with monoclonal actin antibody. Boxed areas outline actin labeling at the active zone (thick arrow) and at the presynaptic membrane outside the active zone (PM outside active zone). Box c and box d are enlarged in panel c and d, respectively. ax, axoplasmic matrix; sv, synaptic vesicles; d, dendrite; g, glia; dp, dense projection.

(c-d) Box c and d in panel b are shown at higher magnification in panel c and d, respectively. Note higher density of gold particles at the synaptic active zone (c) than at the presynaptic membrane outside the synaptic area (d). Thin arrows point at gold particles.

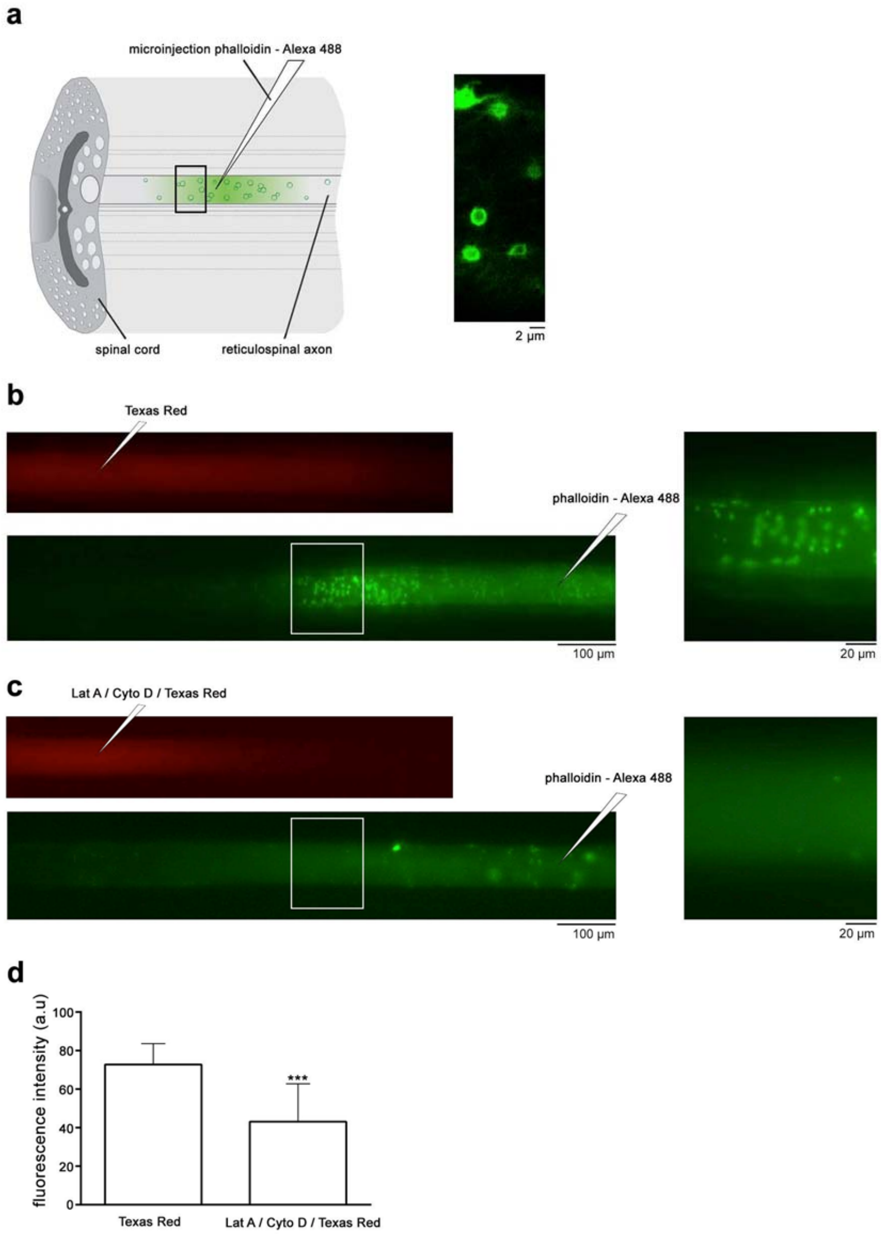

Supplementary Figure 14. Lat A/Cyto D reduces F-actin at giant lamprey axon

(a) Left: A schematic drawing illustrating microinjection of phalloidin-Alexa 488 into a reticulospinal axon. The boxed area marks an area where we examined the injected phalloidin-Alexa 488 fluorescence with a confocal microscope, as shown in the right.
Right: Confocal optical section shows the surface of a living axon injected with phalloidin-Alexa 488 and imaged with a 100x/1.0 NA objective. Each spot of phalloidin-Alexa 488 fluorescence indicates a F-actin spot. The results are consistent with a previous work[1] showing that each F-actin spot corresponds to an active zone and its corresponding periactive zone.

(b) Control experiment: the axon was stimulated at 5 Hz and microinjected first with Texas Red only (red channel). 10 min later, phalloidin-Alexa 488 (green channel) was injected into the same axon ~800 µm away. CCD camera images of the axon were collected with a 10x air

objective at 10-15 min after phalloidin-Alexa 488 injection (left). The boxed area was imaged again, but with a 40x water immersion objective (0.8 N.A.) and shown in the right.

(c) Similar to panel b, except that Texas Red was replaced with Lat A/Cyto D plus Texas Red. The phalloidin-Alexa 488 puncta numbers and intensity were less than panel b.

(d) Bar graph showing a reduction in fluorescence intensity (mean + s.e.m., arbitrary units, a.u.) of the phalloidin-Alexa 488 spots (F-actin spots) in the presence of Texas Red alone (control, 29 spots, 2 axons) or in the presence of Lat A/Cyto D and Texas Red (15 spots, 2 axons). The data were taken from the box area described in panels b and c, ~300-500 µm from the phalloidin-Alexa 488 injection site. ***$P<0.001$, two-tailed Student's t-test.

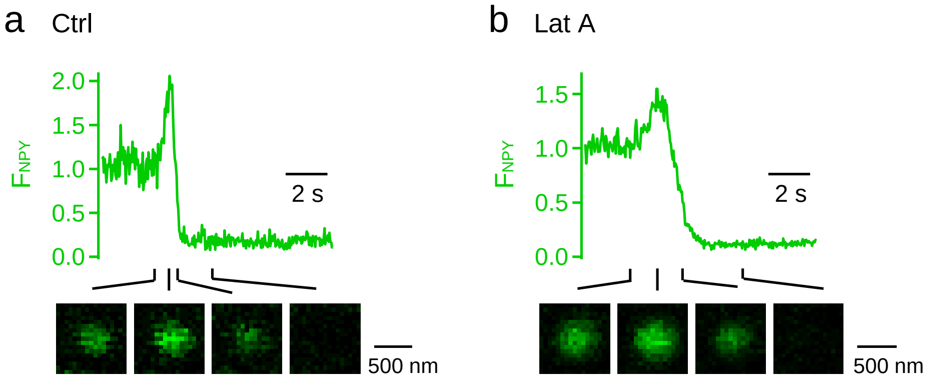

Supplementary Figure 15. Lat A increases the duration of NPY-EGFP release in chromaffin cells

(a-b) Sampled release time course of NPY-EGFP fluorescence (F_{NPY}) in control (a) and in the presence of Lat A (b). The NPY-EGFP images at times indicated are also shown. The initial increase of NPY-EGFP fluorescence is due to fusion pore opening that increases the vesicular lumen pH. The stimulation was $Train_{2Hz}$.

Conclusion: The NPY-EGFP release time, measured as the time at which NPY-EGFP spot fluorescence deceased by 50%, was significantly longer in the presence of Lat A (1.3 ± 0.2 s, n = 84 spots, 27 cells) than in control (0.8 ± 0.1 s, n = 80 spots, 21 cells) (unpaired t-test, $P = 0.013$).

Supplementary References

1. Evergren,E. *et al.* Intersectin is a negative regulator of dynamin recruitment to the synaptic endocytic zone in the central synapse. *J. Neurosci.* **27**, 379-390 (2007).
